# Refining the optimal CAF cluster marker for predicting TME-dependent survival expectancy and treatment benefits in NSCLC patients

**DOI:** 10.1038/s41598-024-55375-0

**Published:** 2024-07-21

**Authors:** Kai Li, Rui Wang, Guo-Wei Liu, Zi-Yang Peng, Ji-Chang Wang, Guo-Dong Xiao, Shou-Ching Tang, Ning Du, Jia Zhang, Jing Zhang, Hong Ren, Xin Sun, Yi-Ping Yang, Da-Peng Liu

**Affiliations:** 1 Department of Otorhinolaryngology‑Head and Neck Surgery, The First Affiliated Hospital of Xi’an Jiaotong University, Xi’an, 710061 Shaanxi China; 2 Department of Thoracic Surgery and Oncology, Cancer Centre, The First Affiliated Hospital of Xi’an Jiaotong University, 277 Yanta West Road, Xi’an, 710061 Shaanxi China; 3 Department of Thoracic Surgery, Qinghai Provincial People’s Hospital, Gonghe Road No. 2, Chengdong District, Xining, 810007 Qinghai China; 4 School of Future Technology, National Local Joint Engineering Research Center for Precision Surgery and Regenerative Medicine, Xi’an Jiaotong University, Xi’an, 710061 Shaanxi China; 5 Department of Vascular Surgery, First Affiliated Hospital of Xi’an Jiaotong University, Xi’an, 710061 Shaanxi China; 6 Oncology Department, The First Affiliated Hospital of Zhengzhou University, Zheng Zhou, 450052 Henan China; 7 Section of Hematology Oncology, Department of Internal Medicine, LSUHSC Cancer Center, School of Medicine, 1700 Tulane Avenue, New Orleans, LA 70112 USA; 8 Department of Pathology, Anatomy and Cell Biology, Sidney Kimmel Cancer Center, Thomas Jefferson University, Philadelphia, PA 19107 USA; 9 Department of Radiotherapy, Shaanxi Provincial Tumor Hospital, 309 Yanta W Rd, Yanta District, Xi’an, 710063 Shaanxi China

**Keywords:** Tumor microenvironment, Cancer associated fibroblasts, scRNA sequence data, Marker selection, Therapy response, Cancer, Immunology, Oncology

## Abstract

The tumor microenvironment (TME) plays a pivotal role in the onset, progression, and treatment response of cancer. Among the various components of the TME, cancer-associated fibroblasts (CAFs) are key regulators of both immune and non-immune cellular functions. Leveraging single-cell RNA sequencing (scRNA) data, we have uncovered previously hidden and promising roles within this specific CAF subgroup, paving the way for its clinical application. However, several critical questions persist, primarily stemming from the heterogeneous nature of CAFs and the use of different fibroblast markers in various sample analyses, causing confusion and hindrance in their clinical implementation. In this groundbreaking study, we have systematically screened multiple databases to identify the most robust marker for distinguishing CAFs in lung cancer, with a particular focus on their potential use in early diagnosis, staging, and treatment response evaluation. Our investigation revealed that COL1A1, COL1A2, FAP, and PDGFRA are effective markers for characterizing CAF subgroups in most lung adenocarcinoma datasets. Through comprehensive analysis of treatment responses, we determined that COL1A1 stands out as the most effective indicator among all CAF markers. COL1A1 not only deciphers the TME signatures related to CAFs but also demonstrates a highly sensitive and specific correlation with treatment responses and multiple survival outcomes. For the first time, we have unveiled the distinct roles played by clusters of CAF markers in differentiating various TME groups. Our findings confirm the sensitive and unique contributions of CAFs to the responses of multiple lung cancer therapies. These insights significantly enhance our understanding of TME functions and drive the translational application of extensive scRNA sequence results. COL1A1 emerges as the most sensitive and specific marker for defining CAF subgroups in scRNA analysis. The CAF ratios represented by COL1A1 can potentially serve as a reliable predictor of treatment responses in clinical practice, thus providing valuable insights into the influential roles of TME components. This research marks a crucial step forward in revolutionizing our approach to cancer diagnosis and treatment.

## Introduction

Lung cancer treatments have been evolving greatly due to targeted therapy and immune therapy, but most patient still have to face the problem of resistance and recurrence ^1,2^. Two important objectives will help to save more lives: (1) early and precise diagnosis, (2) effective and timely therapy evaluation. Researchers have been exploring the aberrant genes signatures for decades, but still not a single marker was confirmed for valuable roles of disease diagnosing and therapy predicating^3^. To define one sensitive candidate, we shifted our focus from cancer cells themselves to tumor microenvironment (TME) component.

TME in lung cancer is a complex network of cells, extracellular matrix, and signaling molecules that interact with cancer cells and play a critical role in tumor growth, invasion, and metastasis ^4,5^. The importance of the TME in lung cancer lies in its ability to shape the behavior of cancer cells and contribute to the development of drug resistance ^6^. One of the key components of the TME in lung cancer is the immune system. Immune cells such as T cells, B cells, and natural killer cells play an important role in recognizing and eliminating cancer cells^7^. However, lung cancer cells have developed mechanisms to evade immune surveillance, leading to immune suppression within the TME. This results in an immunosuppressive TME that allows cancer cells to grow and proliferate.

In addition to immune cells, other stromal cells such as cancer-associated fibroblasts (CAFs) and endothelial cells also contribute to the TME in lung cancer ^8,9^. CAF is a type of stromal cell that is found in the tumor microenvironment (TME) of many types of cancer^10^, and these cells have been shown to play a critical role in the development and progression of lung cancer, and as such, they have become an important target for cancer therapy ^11^. One of the main functions of CAFs in lung cancer is to promote tumor growth and survival by secreting growth factors and other signaling molecules that stimulate the growth and division of cancer cells^4,12,13^. CAFs also help to create a supportive environment for cancer cells by secreting extracellular matrix components that provide structural support for the tumor^14^. In addition to promoting tumor growth, CAFs also contribute to the invasive and metastatic behavior of lung cancer cells, remodeling the extracellular matrix and creating a path for cancer cells to move through^15^. CAFs also secrete enzymes that degrade the surrounding tissue, allowing cancer cells to invade nearby tissues and organs^5,16^.

Until recently, CAFs were noticed to play a role in the development of drug resistance in lung cancer, and CAFs can also promote the development of myeloid-derived suppressor cells (MDSCs), which are a type of immune cell that can suppress the function of T cells and other immune cells ^17^. MDSCs have been shown to contribute to the resistance to immunotherapy in some cancer patients, and some studies have also shown that targeting CAFs can enhance the response to immune therapy in lung cancer ^18^. Meanwhile, CAFs were proved to promote the activation of signaling pathways such as the PI3K-AKT-mTOR pathway, which can lead to resistance to EGFR TKIs in lung cancer ^19,20^. Inhibiting CAFs or targeting these signaling pathways can overcome drug resistance and improve the efficacy of TKI therapy in lung cancer.

With a wealth of research on cancer-associated fibroblasts and their role in the tumor microenvironment, our understanding of the significance of CAF has significantly advanced in laboratory settings^21^. However, the translation of this knowledge into clinical practice remains largely uncharted territory. Moreover, due to limitations in detection methods, harnessing a cluster of CAF markers to identify specific CAF subgroups and utilize these marker signatures for predicting patient survival and treatment responses has been a challenge. Based on the massive studies referring to CAF and TME functional assessment, we began to know the crucial roles of CAF from bench work, but how will it work in clinical practice is now well illustrated. In this pioneering study, we have undertaken a comprehensive exploration of various single-cell databases to identify the most robust fibroblast marker, shedding light on the mechanistic and functional roles of CAF in predicting survival and immunotherapy responses. These novel and promising findings promise to offer a more reliable and trustworthy method for clinical detection and subsequent treatment strategies.

## Methods and materials

### Cancer-associated fibroblasts and control fibroblast groups

The CAF of Human Liver CAFs (CAF113, Human Primary CAFs), Human Breast CAFs (CAF116, Human Primary CAFs), Human Lung Squamous Cell Carcinoma CAFs (CAF117S, Human Primary CAFs), Human Lung Adenocarcinoma CAFs (CAF117, Human Primary CAFs), are cultured for testing the markers. The cancer associated fibroblasts are isolated from human lung tumor, breast tumor, and liver tumor tissues respectively, cells at passage 1 are detached from flasks and immediately cryo-preserved in vials (Each vial contains at least 1,000,000 cells), in this form the cells were shipped to our lab. Cells are used for RNA and protein detection within expanding for five passages at most. In addition to cells culturing, the CAF growth medium was used for limited cells expansion (CAFM03), specifically designed and contained Glucose (Low Glucose), Glutamine (l-Glutamine), HEPES Buffer (HEPES), Phenol Red Indicator (Phenol Red), Puromycin (5 μg/ml).

As to the normal fibroblasts (not activated to become CAF), the control groups of fibroblasts were purchased from ATCC and cultured, and the primary fibroblasts of primary normal Lung fibroblast (PCS-201-013, PCS-201-015) were passed in limited passages, with using recommended medium (Fibroblast Basal Medium, PCS-201-030), serum (Fibroblast Growth Kit-Low serum, PCS-201-041). The trypsin digested procedure shared the same protocol with normal cell culture.

### Western blots

Western blot analysis was described by previous study^22^. Blots were cut prior to hybridisation with antibodies during blotting. The primary antibodies were as follows: anti-COL1A1 (#72026, CST), anti-ACTA2 (ab5694, Abcam), anti-PDGFRA (ab203491, Abcam), anti-VCAM1 (ab174279, Abcam), anti-beta-ACTIN (ab8226, Abcam).

### Single cells RNA sequencing analysis and data processing

The single cell RNA sequencing data was acquired at “IMMUSC-VUE” (IMMUcan Single-Cell RNAseq Database), and “Cell Marker Search”^23^. The TME components deciphering data was obtained at “TIMEDB” ^24^, “TISIDB”^24^. The data and figures were generated automatically online, or generated by analyzing the raw-data, of which the detailed information was described in either results section or in the corresponding figure legends. We achieved hundreds of human lung tissues and lung disease tissues at “Cellxgene”. Specifically, we utilized “CZ CELLxGENE Discover Census” (Chan Zuckerberg Initiative. (n.d.). CZ CELLxGENE Discover. Retrieved, from https://cellxgene.cziscience.com/) to analyze the data, perform Python and R language-based analysis.

### Survival analysis tools and methodology

In addition to information from the single-cell sequencing database, we utilized the KM-plotter online survival analysis tool for clinical indicators such as survival analysis and prognostic analysis^25^. KM-plotter stands out as an advanced online survival analysis tool that performs real-time calculations instead of loading pre-calculated images. The underlying database is meticulously curated manually, incorporating gene expression data, relapse-free, and overall survival information obtained from GEO, EGA, and TCGA. The database is managed by a PostgreSQL server, seamlessly integrating gene expression and clinical data.

To assess the prognostic significance of a specific gene, patient samples are stratified into two groups based on various quantile expressions of the proposed biomarker. A Kaplan–Meier survival plot is employed to compare the two patient cohorts, and the hazard ratio, along with 95% confidence intervals and logrank P-value, is computed. Regular supervision and updates are conducted for both databases and clinical data to ensure accuracy and comprehensiveness. By selecting the target gene and conducting filtering analyses, we can discern differences in disease-free survival, overall survival, and explore variations in clinical survival indicators across different population subsets.

### In vivo study

All mouse studies were approved by the Institutional Animal Care and Use Committee at Massachusetts General Hospital in accordance with institutional guidelines. For generating tumor bearing mouse models, ten million lung cancer cells of A549 were injected subcutaneously with or without fibroblast cells in a 1:2 ratio into flanks of 6–8 weeks old athymic nude mice. In this study, no other interference was given.

### Statistical analysis

Survival analysis is used to estimate the probability of survival or disease progression over time, and to identify factors that may affect these outcomes. Regression analysis is used to model the relationship between one or more predictor variables and an outcome variable. This method is used to identify factors that are associated with lung cancer incidence, progression, or response to treatment. ANOVA is used to compare the expression of genes, proteins, or other biomarkers between different groups of lung cancer patients. Correlation analysis is used to identify biomarkers that are correlated with lung cancer incidence, progression, or treatment response.

### Ethics approval and consent to participate

All procedures performed in studies involving animals and human participants (including the use of tissue samples) were in accordance with the ethical standards of the institution or practice at which the studies were conducted. We stated that the protocol for the research project has been approved by the Ethics Committee of the First Affiliated Hospital of Xi’an Jiaotong University, and that it conforms to the provisions of the Declaration of Helsinki. We stated that the protocol adheres to the ARRIVE guidelines for the reporting of animal experiments. We stated that informed consent was obtained from all subjects and/or their legal guardian.

## Results

### Cancer associated fibroblasts constituted the important part of TME

The tumor microenvironment (TME) comprises various functional subgroups of both immune and non-immune cells. The intricate regulatory interactions within this environment are primarily orchestrated by mediators found in the cluster of cancer-associated fibroblasts (CAFs). To establish the presence and functional roles of CAFs, we initially conducted examinations of stromal cell staining and analyzed single-cell RNA (scRNA) datasets. Our investigations revealed that fibroblasts are a prominent component of lung tissues (see Fig. S1A). Further scrutiny demonstrated that CAFs represent a significant proportion of both lung adenocarcinoma (LUAD, Fig. S1B) and lung squamous carcinoma (LUSC, Fig. S1C). Our validation extended to single-cell analysis databases, where we observed abundant enrichment of fibroblasts in GSE85716 (Fig. S1D), GSE118370 (Fig. S1E), and in two TCGA lung cancer datasets (Fig. S1F,G). The further deciphering of GSE85716 dataset proved that although different analyzing modes correlates with different CAF ratios, CAF was always the important and main component (Fig. S2).

### Define the cancer associated fibroblasts with most effective marker

**Figure 1:**
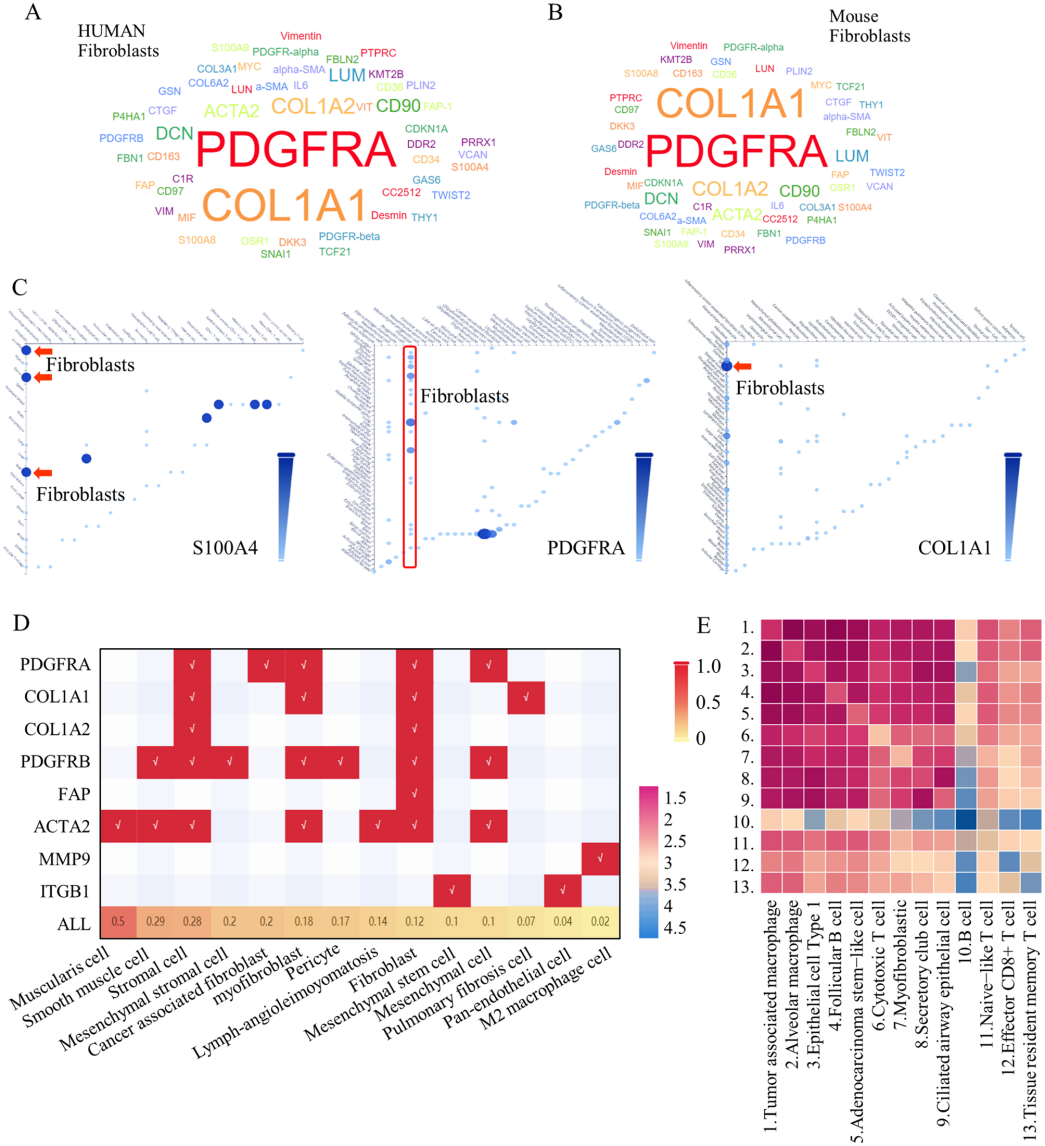
The markers mostly used to define the subgroup of TME fibroblast. Through screening and test on website of “Surface markers”, the most accepted and universally used fibroblasts in human (**A**) and in mouse (**B**) were displayed. (**C**) The markers of COL1A1 and PDGFRA showed the best specificity. (**D**) Plotting results indicated that COL1A1, COL1A2, and FAP showed best representation. (**E**) Fibroblasts showed close connections with other kinds of TME subgroups in GSE lung cancer samples, and each number indicated one special subgroup as was labeled (raw data could be achieved at http://117.50.127.228/CellMarker/CellMarker_communication.jsp).

Fibroblasts constitute a pivotal element within the TME groups, and numerous signature markers have been identified and partially validated in both human (Fig. 1A) and mouse (Fig. 1B) models. Among these markers, COL1A1 and PDGFRA have emerged as the most widely recognized and universally accepted, demonstrating the highest specificity (Fig. 1C). A plot map consisting all fibroblasts markers are enrolled to test their expressing patterns, more specifically, COL1A1, COL1A2, and FAP showed best correlation (Fig. 1D), and fibroblasts, myofibroblasts included, showed close connection with other kinds of TME subgroups (Fig. 1E, Fig. S3A,B).

To elucidate the distinct characteristics of each potential CAF marker within all TME groups, we meticulously examined single-cell RNA sequencing data from various human organs and systems at the Tubular Institute. This analysis unveiled diverse distribution patterns among different TME subgroups (see Fig. S4A). Upon closer examination, it became evident that many fibroblast markers exhibited limited specificity in identifying unique subgroups (Fig. S4B, C). Furthermore, universal markers such as COL1A1, ITGB1, PDGFRA, PDGFRB, FAP, SOX4, NOTCH3, KLF4, ACTA2, and S100A4 displayed either limited or non-specific distribution in the comprehensive organ-wide analysis. However, we observed that ITGB1, COL1A1, and S100A4 exhibited high sensitivity, while COL1A1, PDGFRA, PDGFRB, and FAP demonstrated exceptional specificity (Fig. S4D, E).

### Expression profiling of CAF markers in lung cancer stromal cell groups

**Figure 2:**
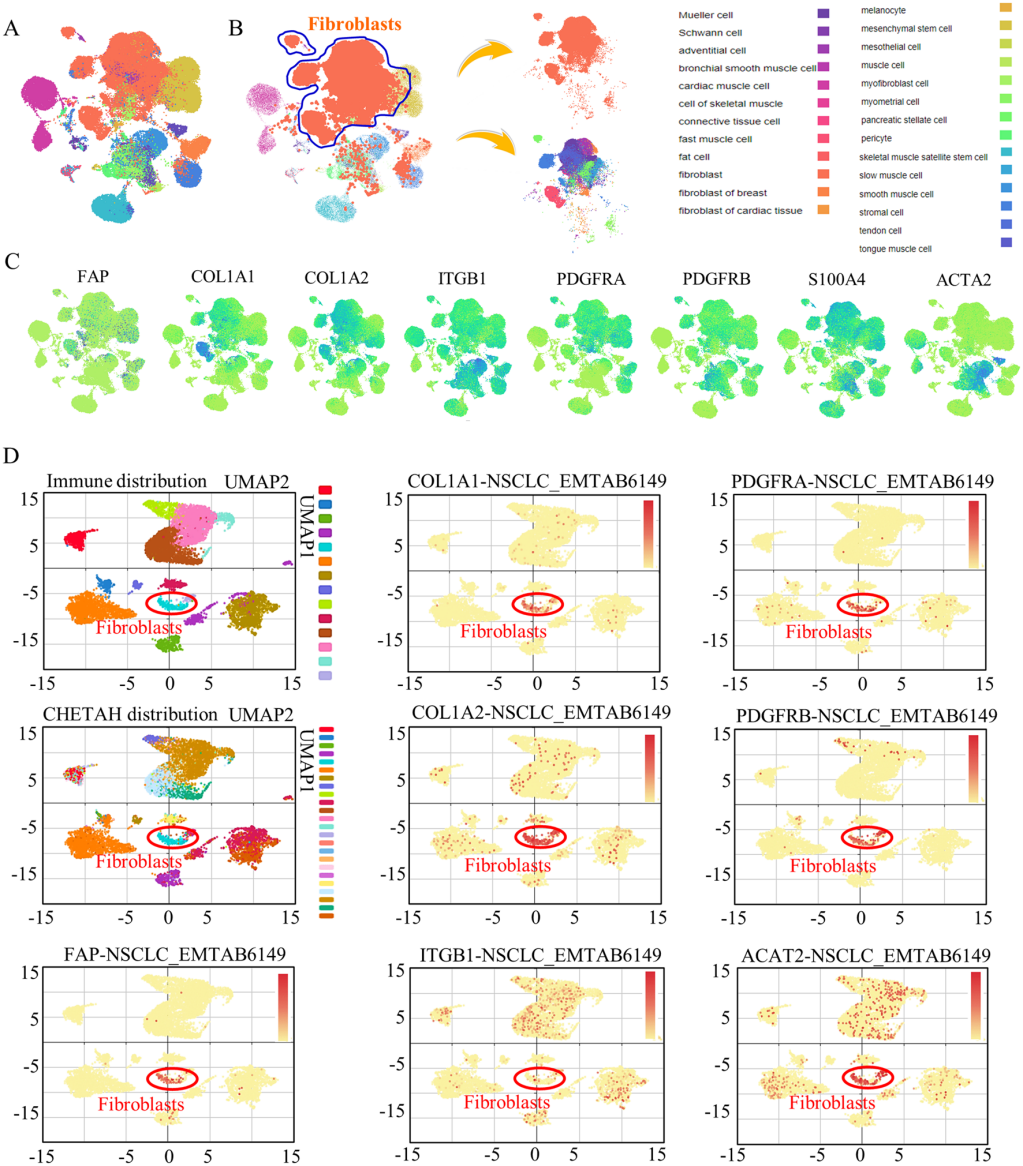
The expression patterns of CAF markers in pan-cancer and lung cancer. (**A,B**) Stromal cells from all organs were enrolled for identify the fibroblasts markers, and the distribution of fibroblasts were labeled with dark purple plots. (**C**) Different CAF markers showed diverse expression motif and distribution patterns. Lung tissues were divided into different subtypes (**D**), and in cluster of c-9 of fibroblasts, COL1A1, COL1A2, FAP, and PDGFRA showed stronger expressions and better cluster enrichment.

In a more detailed examination, we focused on fibroblasts derived from various human tissues. Our investigation established that the majority of fibroblasts are indeed among the stromal cell population (see Fig. 2A, B). We further verified the sensitivity and specificity of FAP, COL1A1, and COL1A2 in identifying fibroblasts. The significantly heightened expression of these markers unequivocally correlated with the proportional representation of both fibroblasts and cancer-associated fibroblasts (CAFs) (Fig. 2C). Further in lung cancer tissues, through scRNA analysis at “IMMUcan SingleCell RNAseq-Database” of lung cancer, for the first time, we proved the specificity of COL1A1, COL1A2, and FAP, all of three are highly enriched in subgroup of CAF (Fig. 2D), the cluster of which was circled. While other CAF markers were either lowly expressed, or less enriched. Among all these possible candidate markers, FAP, is predominantly enriched in cancer-associated fibroblasts. Further in lung cancer groups, scRNA analysis showed higher expressing levels of COL1A1M COL1A2, PDGFRA and PDGFRB in identifying the CAF clusters specifically (Fig. S5).

### The solid CAF markers are associated with different malignant signatures

**Figure 3:**
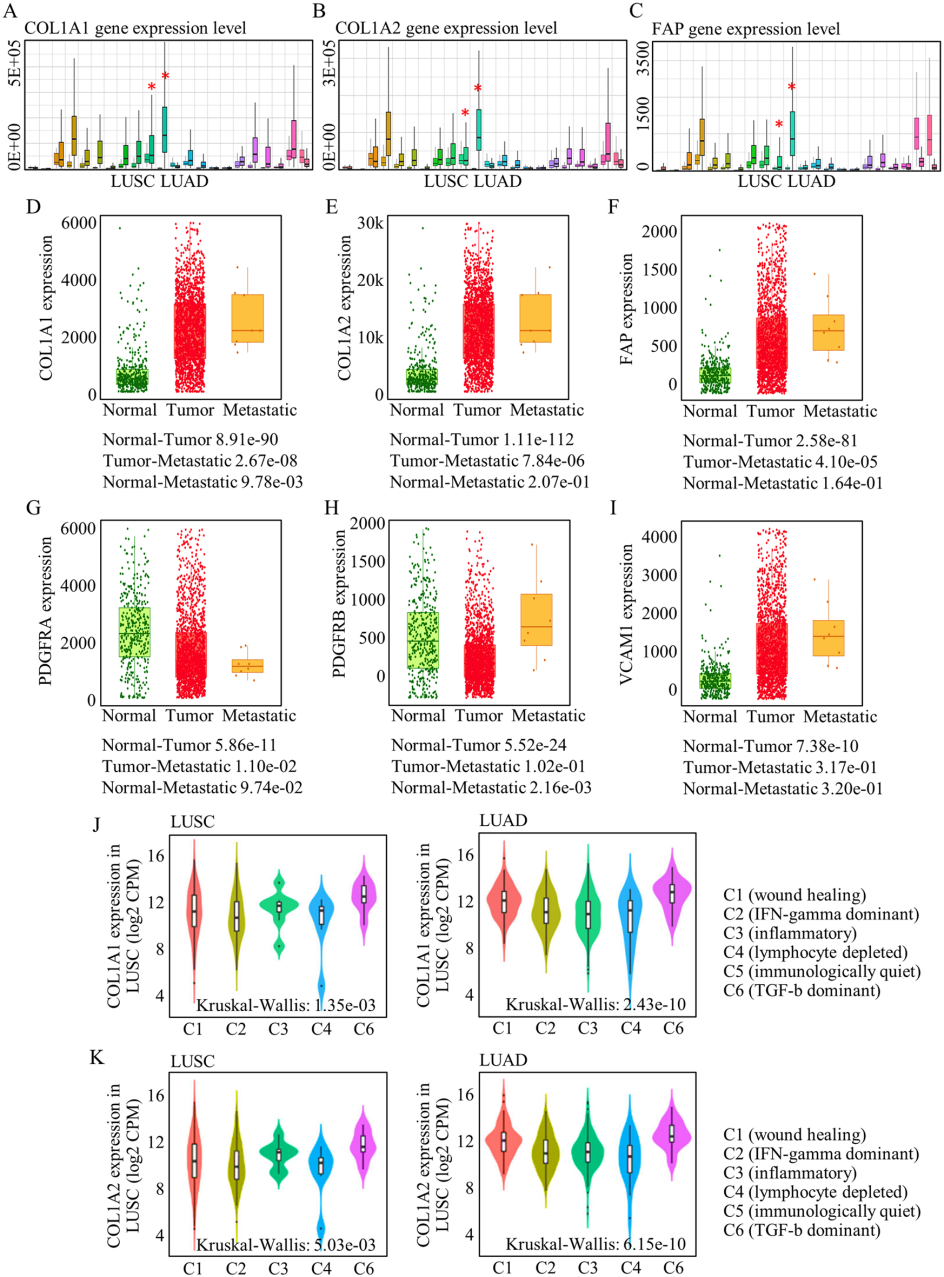
CAF correlated with tumor group and indicated malignancy status. The markers of COL1A1 (**A**), COL1A2 (**B**), and FAP (**C**) are significantly highly expressed in lung cancer. COL1A1 (**D**), COL1A2 (**E**), and FAP (**F**) indicated a higher chance of metastasis. (**G–I**) The CAF markers of PDGFRA, PDGFRB, and VCAM1 did not successfully differentiate tumor group and normal tissues, or did not distinguish metastatic sites. (**J,K**) COL1A1 and COL1A2 were differently enriched in different cluster, and their enrichment were much stronger in c-6 group that can most prominently represent CAF function.

Prior investigations have yet to explore the predictive potential of CAF markers for discerning malignancies and metastatic conditions. This gap in knowledge has been exacerbated by the lack of efficient cell isolation methods and a shortage of highly specific markers. Leveraging our systematic analysis of various markers, we have identified COL1A1, COL1A2, FAP, PDGFRA, and PDGFRB as candidates for their roles in distinguishing malignancies from normal tissues. The markers of COL1A1 (Fig. 3A), COL1A2 (Fig. 3B), and FAP (Fig. 3C) are significantly highly expressed in lung cancer, compared to normal tissues, while PDGFRA, PDGFRB, and ACTA2 either failed to be differentially expressed in cancer tissues, or were expressed in relative lower levels (Fig. S7A-C). More specifically and importantly, COL1A1 (Fig. 3D), COL1A2 (Fig. 3E), and FAP (Fig. 3F) were all highly expressed in metastatic cancer tissues, possibly indicated a higher chance of metastasis when abnormally expressed, which have not been reported before. However, they were closely related to CAF-based TME functions referring to carcinogenesis and tumor metastasis.

In contrast, other potentially identified CAF markers did not exhibit the capacity to discriminate between tumors, and their contributions to assessing survival outcomes remained unclear (see Fig. 3G–I). Consequently, we used COL1A1 and COL1A2 as CAF markers for assessing immune-related cells functions using the public dataset of “TISIDB”. Notably, their enrichment was most pronounced in the c-6 group, which signifies the predominant representation of CAF functionality (see Fig. 3J, K).

### Survival predicating roles of the representative COL1A1, COL1A2, and FAP

To define the roles of each subgroup of CAF population, we applied scRNA analysis of two TCGA datasets (Fig. S6A, B), and fibroblasts constituted the majority portion in each LUAD sample dataset (Fig. S6C). To delve deeper into our analysis, we proceeded to examine the sub-clone clusters among lung cancer patients. We classified fibroblasts based on their respective immune-regulator expression patterns on two TCGA datasets (see Fig. S6D, E). It became evident that distinct fibroblast clusters were associated with varying progression and overall survival outcomes. Given the diversity in the representative roles of different CAF markers, we subsequently employed single markers to investigate their diagnostic and predictive capabilities.

**Figure 4:**
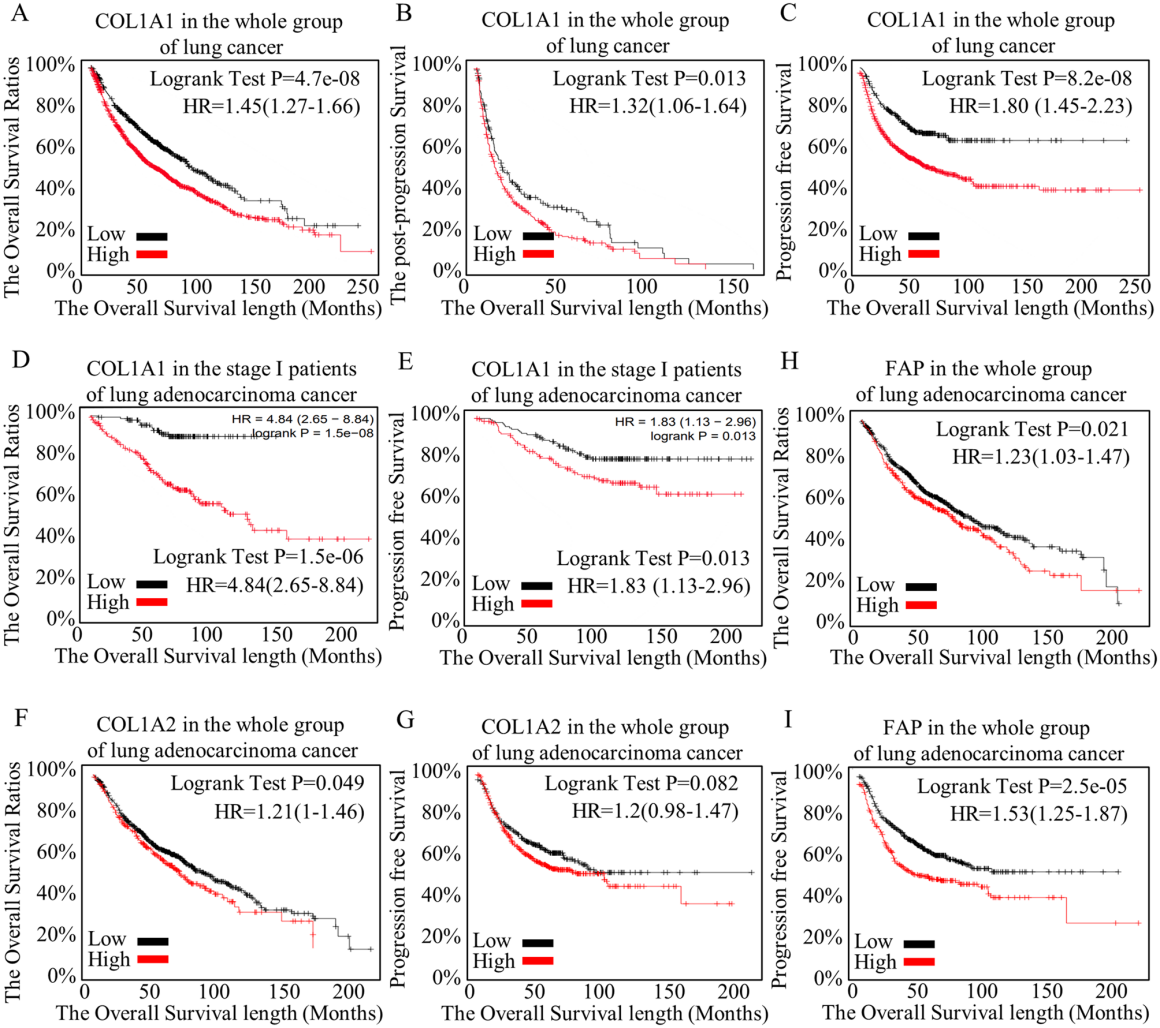
Survival predicating roles of the representative COL1A1, COL1A2, and FAP. As the specific CAF marker could distinguish the malignant state of the tumor and identify the presence or absence of metastasis, we further analyzed its role in predicting survival. Higher COL1A1 expression pointed to shorter overall survival (**A**), post-progression survival (**B**), and progression free survival (**C**) in whole cancer groups. In subgroup analysis, COL1A1 greatly helped to defined high-risk groups at early stage (**D,E**). Also, COL1A2 (**F,G**) and FAP (**H,I**) indicated a significant role in differentiating survival expectances. Other factors failed to distinguish differences in survival or differences in disease-free survival.

Given that a specific CAF marker can discern the malignancy of a tumor and determine the presence or absence of metastasis, we conducted a further analysis to examine its predictive capacity for survival. Increased COL1A1 expression was associated with shorter overall survival (Fig. 4A), post-progression survival (Fig. 4B), and progression-free survival (Fig. 4C) across the entire spectrum of cancer patients. In subgroup analysis, COL1A1 proved to be highly valuable in identifying high-risk groups at an early stage (Fig. 4D, E). Furthermore, COL1A2 (Fig. 4F, G) and FAP (Fig. 4H, I) also played significant roles in differentiating survival expectations. Conversely, other factors failed to distinguish differences in survival or variations in disease-free survival. The CAF marker of COL1A1 indicated overall survival and disease-free survival more efficiently in early staged lung adenocarcinoma. COL1A2 did not distinguish the survival differences of overall, progression, and post-progression in the whole groups of lung cancer patients (Fig. S7D), However, it did actually accurately and efficiently in early staged lung adenocarcinoma (Fig. S7D). As to FAP, although it could indicate survival expectance in overall survival, and the progression-free of stage I lung adenocarcinoma, but FAP did not define any difference in progression-free of other groups of lung adenocarcinoma (Fig. S7E).

### CAF ratios correlated with different treatments responses

**Figure 5:**
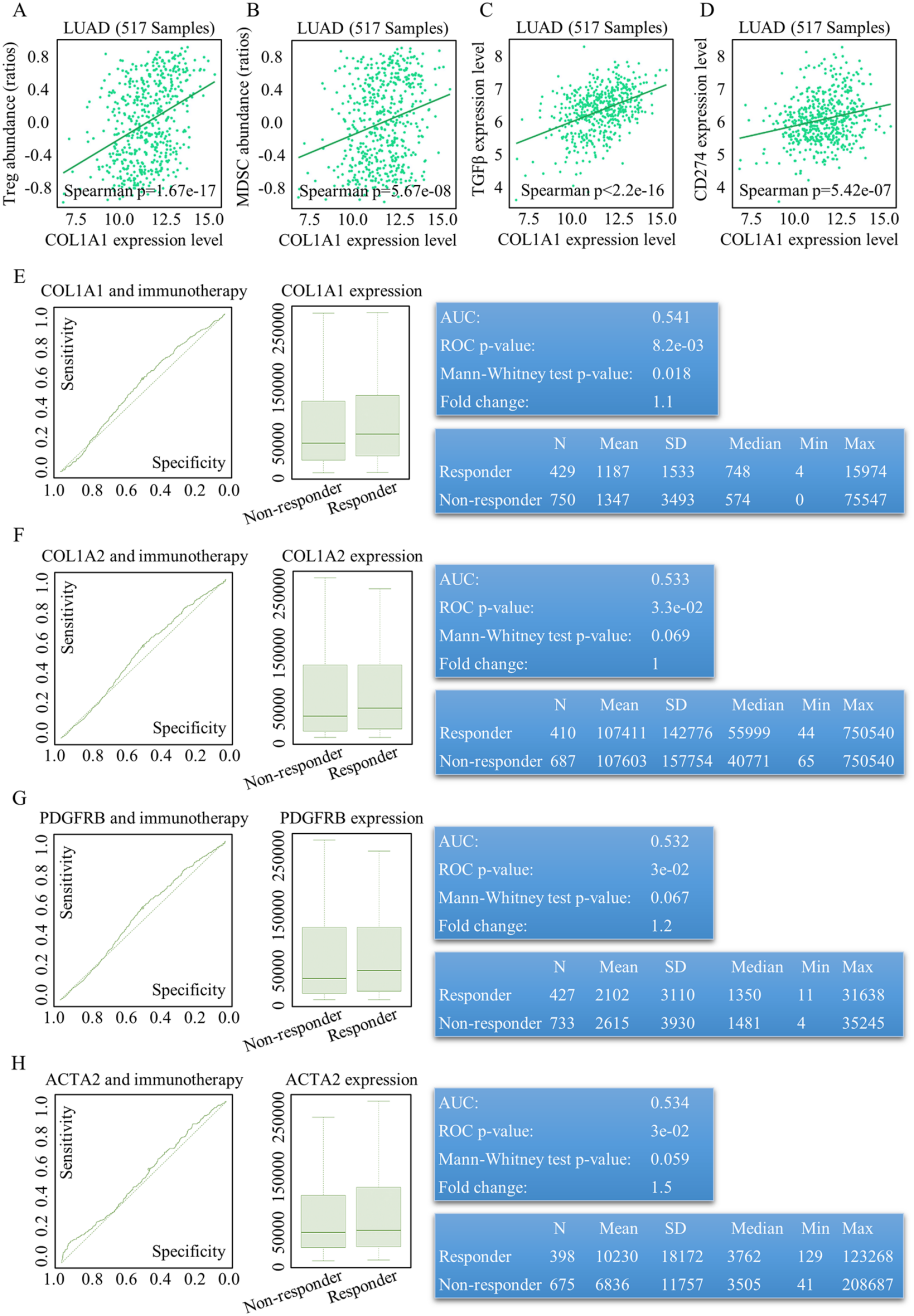
CAF ratios differences indicated different immune therapy response. COL1A1 positively correlated with inhibitory immune cells [(**A,B**), T-regulator ratios, raw data could be acquired at http://cis.hku.hk/TISIDB/data_temp/COL1A1_exp_LUAD_TIL_Treg.txt, and MDSC, raw data could be acquired at http://cis.hku.hk/TISIDB/data_temp/COL1A1_exp_LUAD_TIL_MDSC.txt], secretive CAF functional factors [(**C**), TGFβ expression and secretion, raw data could be acquired at http://cis.hku.hk/TISIDB/data_temp/COL1A1_exp_LUAD_Immunoinhibitor_TGFB1.txt], and negative immune regulator [(**D**), CD274 (PD-L1), raw data could be acquired at http://cis.hku.hk/TISIDB/data_temp/COL1A1_exp_LUAD_Immunoinhibitor_CD274.txt]. Increased CAF markers of COL1A1 (**E**), COL1A2 (**F**), PDGFRB (**G**), ACTA2 (**H**) indicated better immune therapy response.

After a thorough assessment of CAF roles in TME, malignancy defining, survival predication, we finally assessed the ratios and functions of CAF in evaluating the treatment effect. To evaluate the therapy response referring to different CAF markers, we utilized ROC Plotter at https://www.rocplot.com/, and COL1A1 positively correlated with negative immune regulators, including inhibitory immune cells (Fig. 5A, B), secretive CAF functional factors (Fig. 5C), and negative immune regulators (Fig. 5D). In our analysis, we investigated the predictive value of CAF markers in relation to immunotherapies across various malignancies. It was observed that elevated levels of CAF markers, specifically COL1A1 (Fig. 5E), COL1A2 (Fig. 5F), PDGFRB (Fig. 5G), and ACTA2 (Fig. 5H), were all associated with improved responses to immune therapy. Moreover, COL1A1 and COL1A2 also demonstrated superior predictive abilities for the response to TKI (Tyrosine Kinase Inhibitor) treatment, showing practical levels of sensitivity and specificity (see Figs. S8 and S9).

### Identification of the applicable clinical and translational values for CAF subgroup

**Figure 6:**
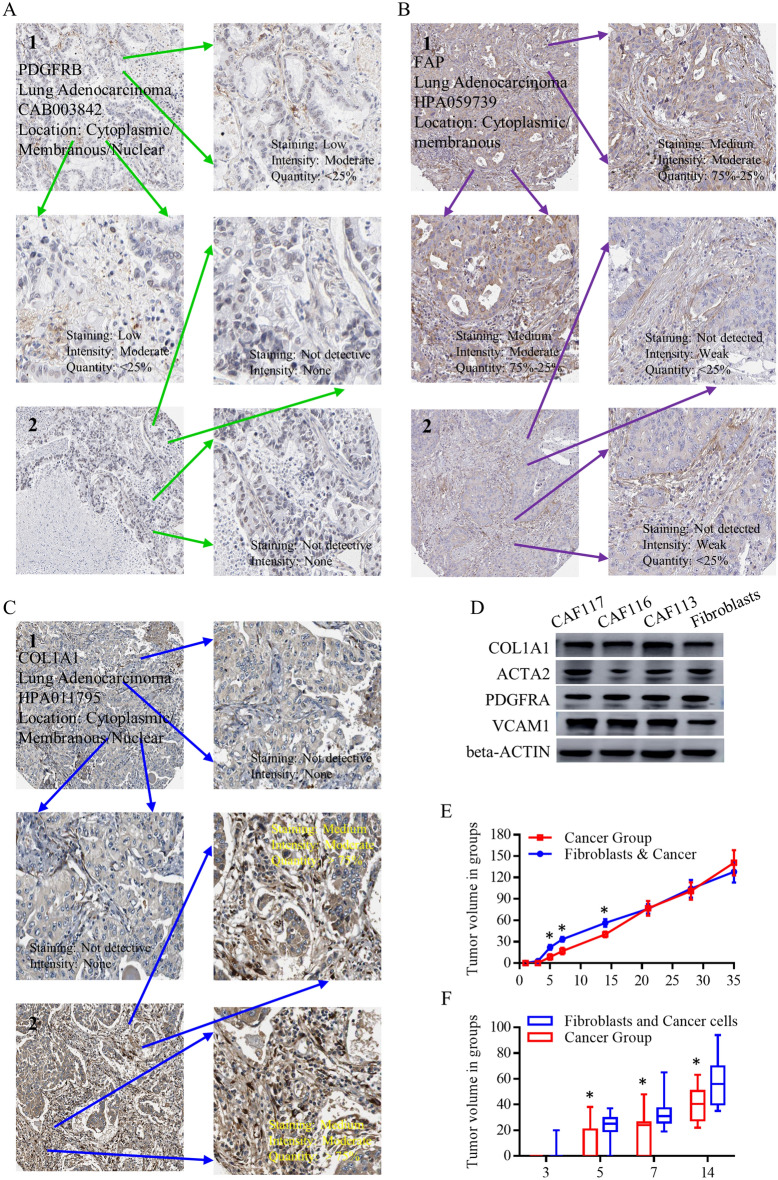
The applicable clinical and translational usage for CAF detections and functions. The IHC results from protein-atlas were screened for checking the expressing patterns of enrolled CAF candidates of PDGFRB (**A**), FAP (**B**), and COL1A1 (**C**). These genes are not universally expressed in lung cancer tissues. (**D**) We applied three cell lines of cancer associated fibroblasts, in addition with control group of fibroblasts, to check the protein levels, and almost every CAF marker could be detected in different groups. (**E**) In vivo study indicated the higher tumor formation ability and highly proliferative ability of tumors triggered by co-embedded CAF groups when performing subcutaneous cancer cells injection.

Numerous scRNA array analyses have shed light on the substantial involvement of CAFs in the composition and functional regulation of the TME. To accurately unravel the potential presence and roles of CAFs and their clinical and translational significance, we initiated our investigation by examining the immunohistochemistry (IHC) results obtained from the Protein Atlas database. These results were scrutinized to assess the expression patterns of the selected CAF candidates, including PDGFRB (Fig. 6A), FAP (Fig. 6B), and COL1A1 (Fig. 6C). These genes are not universally expressed in lung cancer tissues, only implied for the indicative CAF subgroup, and the total expression level was not high, proving their specific expressing patterns. Further, we applied three cell lines of cancer associated fibroblasts, in addition with the control group of fibroblasts, to check the protein levels. As to every CAF marker, it could be detected in different groups, however, with different expressing intensity. PDGFRA and COL1A1 were universally expressed in three commercially cancer associated fibroblasts. To address their specific expressing signatures, we detected the relative CAF markers in lung cancer cell lines, in clinical lung cancer tissues. COL1A1, ACTA2, and alpha-SMA are relatively grouped in lung tissues, and PDGFRA, VCAM1 are expressed in both lung tissues and lung cancer cell lines, showing less specificity (Fig. S10A). When looking into the CAF markers more specifically, COL1A1, ACTA2, and alpha-SMA are not significantly different in cancer tissues, compared to adjacent lung tissues, partially proving their roles may be allocated to the TME subgroup, not in whole cancer tissues or lung tissues. Nude mice implanted with lung cancer cells alone or associated with lung cancer fibroblasts were observed and calculated after injection for 35 days. In vivo study indicated the higher tumor formation ability and highly proliferative ability of tumors triggered by co-embedded CAF groups when performing subcutaneous cancer cells injection (Fig. 6E, F), but no significant endpoint differences were noticed at the 35th day (Fig. S10B–D).

## Discussion

CAFs are a type of stromal cell that plays a critical role in tumor growth and progression, as they are responsible for producing extracellular matrix components and growth factors that promote tumor cell proliferation, migration, and invasion. CAFs are generally transformed from normal fibroblasts, and are closely associated with primary tumor cells and participate in the neoplastic process ^26^. There is reciprocal communication between CAFs and tumor cells through paracrine effects of secreted growth factors, cytokines & chemokines from both fibroblasts, tumor cells and other tumor-associated cells ^16,27^. CAFs are potential target for cancer therapy and enhanced understanding of tumor growth, angiogenesis and metastasis.

The targeted therapy and the immune therapy responsive indicators can vary depending on the type of cancer and the specific treatment being used. There is ongoing research to identify biomarkers that can predict response to either EGFR-TKI therapy and to immune therapy in lung cancer, including gene expression profiling and analysis of immune cell populations within the tumor microenvironment. However, due to limited research methods and understanding of cellular interactions and intracellular interrogating, no single indicator was ever identified to represent the tumor progression or to indicate therapy response. As the TME components, especially the immune regulators predominantly controlled many intercellular communications and the intracellular regulations, we hypothesized the fibroblasts group plays the role of a relay station regulator. Many important studies explored and reported the TME associated CAF functions with several recognized markers, however, many analyzing errors and statistical bias inevitably occurred during scRNA processing, because different digital libraries containing diverse CAF markers regarding a single edited gene-barcode ^28–31^.

We usually utilize the cancer cells markers and functional markers to assess the malignancy and relative therapeutic response. However, the TME component were crucial for cancer group, but their potential roles in evaluating the tumor bulk signatures were neglected^20,32,33^. Cancer-associated fibroblasts (CAFs) play a crucial role in the tumor microenvironment (TME) of lung cancer, but their potential roles in assessing lung cancer patients survival and therapy response were not well illustrated To start, wee confirmed the presence of fibroblasts, including CAFs, through staining and single-cell RNA sequencing (scRNA) analysis of lung tissues (Fig. S1A, B). Additionally, CAFs were found to be the main group in both lung adenocarcinoma (LUAD) and lung squamous carcinoma (LUSC) (Fig. S1C, D). By screening various databases, we consistently observed the abundant enrichment of fibroblasts, particularly CAFs, in lung cancer datasets (Fig. S1E–H, Fig. S2).

To identify effective markers for identifying the CAFs group, we assessed multiple fibroblast markers, and found that COL1A1 and PDGFRA exhibited the best specificity in CAFs, and were selected as potential candidates for assessing their clinical application values (Fig. 1C). We check all related fibroblasts’ markers by using multiple publica datasets and clinical tissues banks. Among all the defined and recognized markers, COL1A1, COL1A2, and FAP showed a strong correlation in their expression patterns, indicating their potential as reliable markers for CAF identification (Fig. 1D). Fibroblasts, including myofibroblasts, displayed close connections with other TME subgroups (Fig. 1E, Fig. S3A, B). Further analysis of scRNA sequencing data from human organs revealed that ITGB1, COL1A1, S100A4, and FAP were highly sensitive markers, while COL1A1, PDGFRA, PDGFRB, and FAP were highly specific for CAFs (Fig. S4D, E). In lung cancer stromal cells, FAP, COL1A1, and COL1A2 exhibited high sensitivity and specificity, reflecting the abundance of fibroblasts and CAFs (Fig. 2C, D, Fig. S5).

The ratios of CAF subgroups were associated with different survival outcomes. Higher proportions of CAF cells were correlated with shorter survival in lung adenocarcinoma (LUAD), particularly when using COL1A1 as a marker (Fig. 4A–D). COL1A2 and FAP also played significant roles in predicting survival outcomes (Fig. 4I–L). However, other CAF markers did not demonstrate distinctive capabilities or survival associations (Fig. 4, TCGA data not shown).

Analyzing the expression patterns of CAF markers in lung cancer tissues compared to normal tissues, we found that COL1A1, COL1A2, and FAP were significantly upregulated, indicating their potential as markers for identifying malignancies and metastatic diseases (Fig. 3A–C). Notably, COL1A1, COL1A2, and FAP were closely associated with CAF-mediated functions related to carcinogenesis and tumor metastasis (Fig. 3D–F). The clinical and translational value of CAF subgroups was supported by protein expression analysis and in vivo studies using cancer-associated fibroblast cell lines (Fig. S9A, Fig. 6E, F). In evaluating the treatment response, COL1A1 positively correlated with negative immune regulators and indicated better responses to immune and TKI therapies (Fig. 5A–H, Figs. S7, S8). Additionally, COL1A2 showed potential as a predictive marker for immune therapy response (Fig. 5F).

While the article provides valuable insights into the role of cancer-associated fibroblasts in the tumor microenvironment of lung cancer and introduces potential markers for CAF identification, there are several limitations and areas for improvement: (1) generalization of findings: the study extensively relies on the analysis of lung cancer datasets, and the generalization of findings to other types of cancer may not be justified. It would be important to investigate the role of CAFs and the identified markers in various cancer types to assess their broader applicability. (2) Statistical bias and analyzing errors: the article mentions that analyzing errors and statistical bias were inevitable during single-cell RNA sequencing processing. It is crucial to address and minimize these biases to enhance the reliability of the findings. Providing more details on the steps taken to mitigate such biases would strengthen the study. (3) Lack of validation in independent cohorts: the study could benefit from validation in independent cohorts to confirm the reproducibility of the results. This would add robustness to the identified markers and their associations with survival outcomes and treatment responses. (4) Limited discussion on functional mechanisms: while the article touches on the clinical and translational value of CAF subgroups, it lacks in-depth exploration and discussion of the functional mechanisms through which these markers influence carcinogenesis, tumor metastasis, and treatment responses. A more detailed mechanistic understanding would enhance the clinical relevance of the findings. (5) Insufficient discussion on heterogeneity: the article does not extensively discuss the heterogeneity of CAFs, which is a critical aspect to consider in understanding their roles in the TME. Addressing CAF heterogeneity could provide a more nuanced understanding of their contributions to tumor progression and treatment responses.

In summary, our study unveils the profound significance of Cancer-Associated Fibroblasts within the Tumor Microenvironment of lung cancer. Notably, the newly identified markers, COL1A1 and COL1A2, stand out as versatile tools for CAF identification, survival prognosis, and the evaluation of treatment responses in lung cancer patients. What sets our research apart is the revelation that CAF markers, hitherto unexplored in this context, exhibit more pronounced effectiveness in predicting lung cancer metastasis, survival, and treatment responses compared to traditional lung cancer markers like CEA, CYFRA21-1, CA199, SCC, and other candidate biomarkers. These unprecedented findings open up new avenues for identifying promising and reliable candidates from the TME components, which have not been previously reported. Ultimately, our study advances our understanding of the pivotal role played by CAFs in lung cancer and carries profound implications for the development of personalized treatment strategies.

## Conclusion

In this groundbreaking study, we delved into the intricate role of cancer-associated fibroblasts within the tumor microenvironment of lung cancer. We successfully pinpointed specific markers, notably COL1A1, COL1A2, and FAP, as key identifiers of CAFs. These markers have the potential to be proved of invaluable, connecting with a range of clinical outcomes such as survival duration, malignancy assessment, metastasis prediction, and treatment response evaluation. This research is notably novel as it shifts the focus from mere cancer group indicators to the potential of CAFs as robust prognostic indicators and promising therapeutic targets. It underscores the pivotal role played by CAFs within the TME components in lung cancer, shedding light on their utility for personalized treatment strategies. However, further investigations are warranted to unravel the underlying mechanisms and validate these remarkable findings.

Moreover, the cluster of CAF markers holds great promise for being designed into a comprehensive detection panel, which can effectively translate TME signatures into clinical practice, thereby bestowing numerous clinical benefits on patients. This study lays the foundation for a new era in the management of lung cancer, where the TME is at the forefront of personalized treatment approaches.

### Supplementary Information

Supplementary Information.

Supplementary Figure S1.

Supplementary Figure S2.

Supplementary Figure S3.

Supplementary Figure S4.

Supplementary Figure S5.

Supplementary Figure S6.

Supplementary Figure S7.

Supplementary Figure S8.

Supplementary Figure S9.

Supplementary Figure S10.

Supplementary Legends.

## Data Availability

The datasets during and/or analyzed during the current study available from the corresponding author on reasonable request.
