# Supplementary material for: Refining the optimal CAF cluster marker for predicting TME-dependent survival expectancy and treatment benefits in NSCLC patients
Source: Sci Rep. 2024 Jul 21;14:16766. doi: 10.1038/s41598-024-55375-0 (PMC11271481; doi:10.1038/s41598-024-55375-0)
Supplement: Supplementary file 1 — Supplementary Information. [file 41598_2024_55375_MOESM1_ESM.pdf]

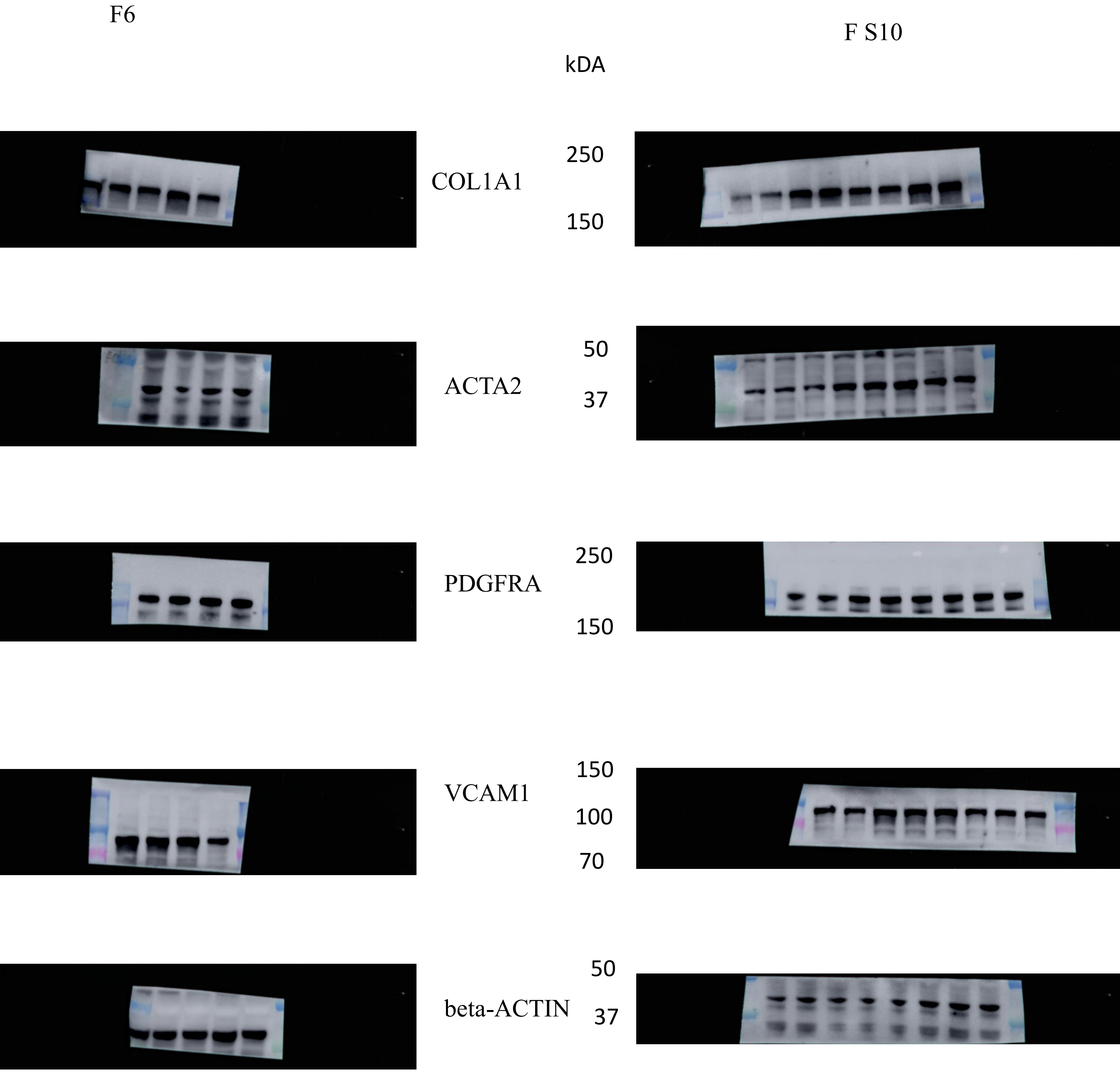
F6
F S10
kDA
250
COL1A1
150
50
ACTA2
37
250
PDGFRA
150
150
VCAM1
100
70
50
beta-ACTIN
37

F6

F S10

COL1A1

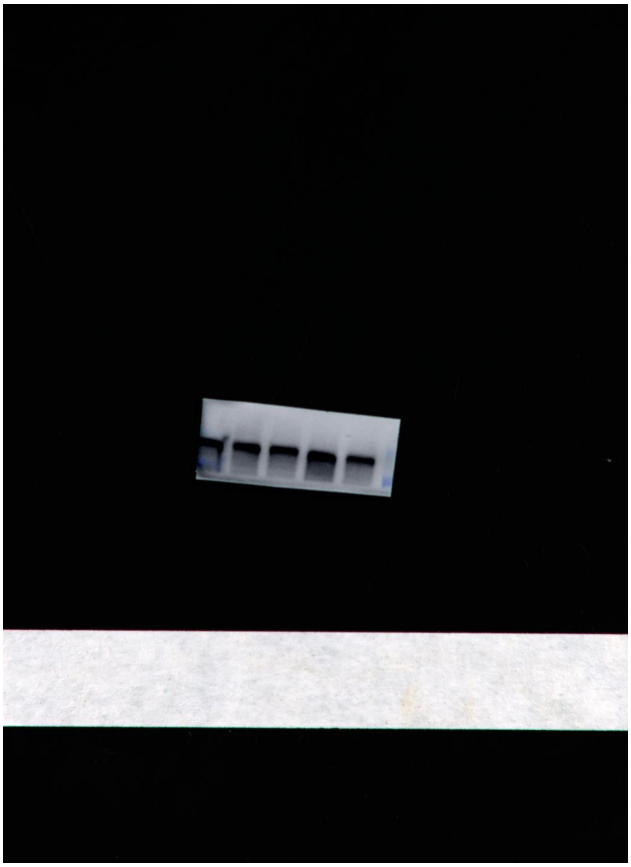

250

150

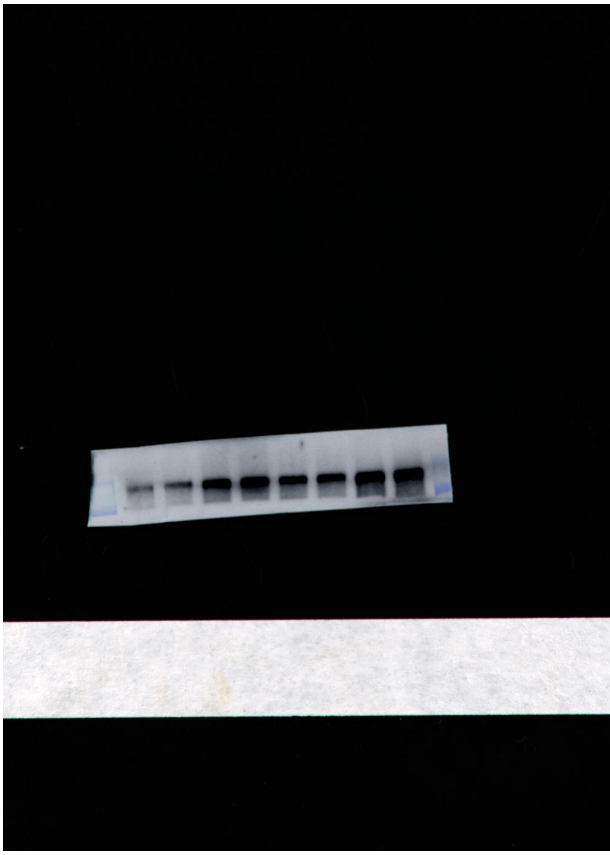

F6

F S10

ACTA2

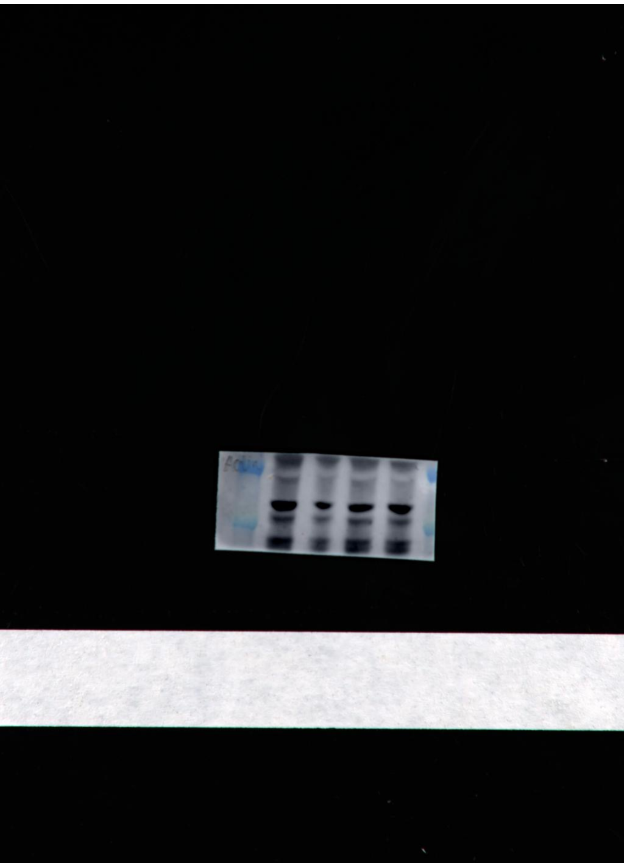

50

37

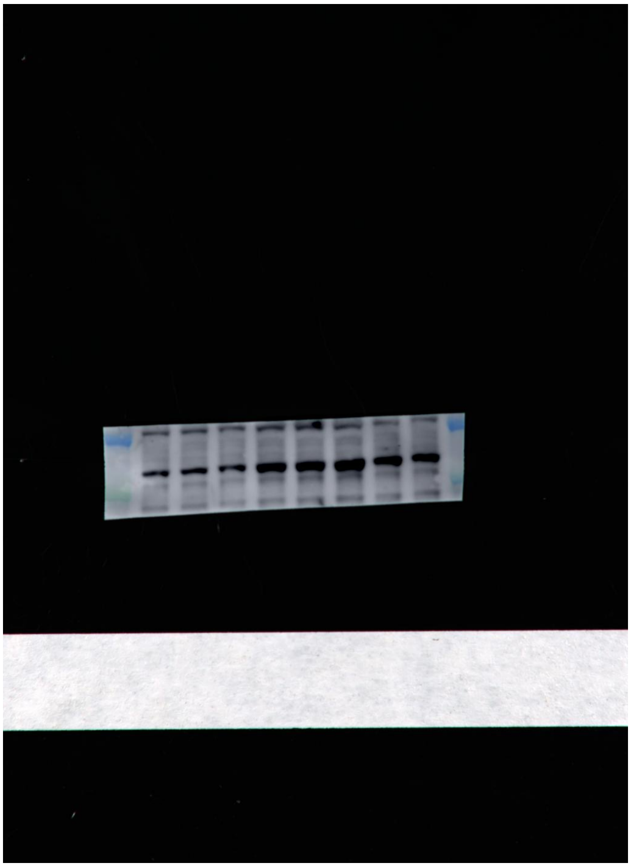

F6

F S10

PDGFRA

[PDGFRA Antibody (PA5-14709) (thermofisher.com)]

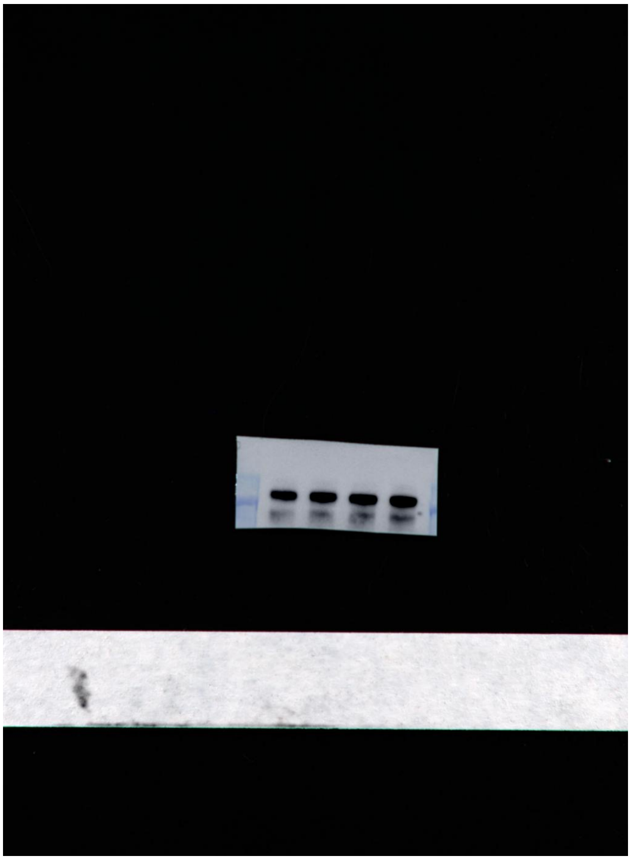

250

150

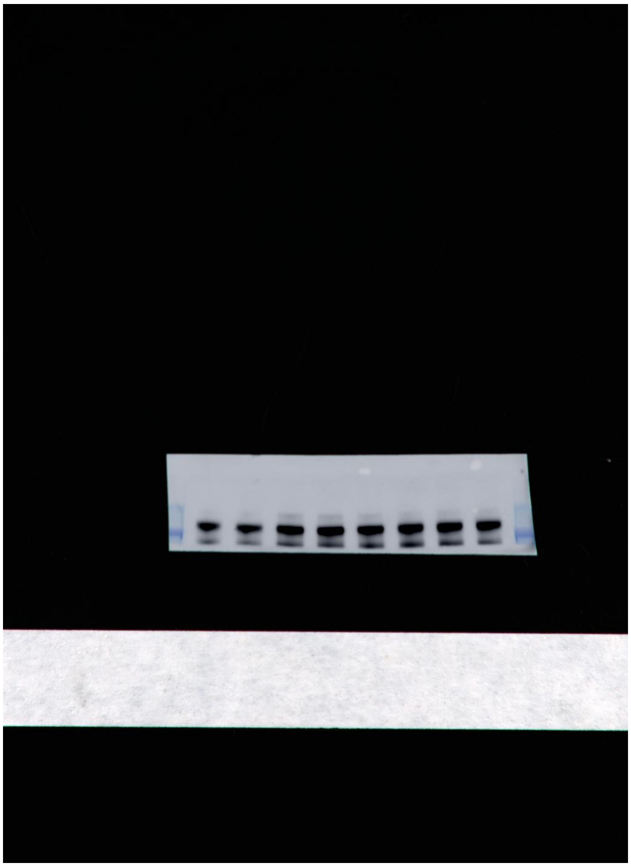

F6

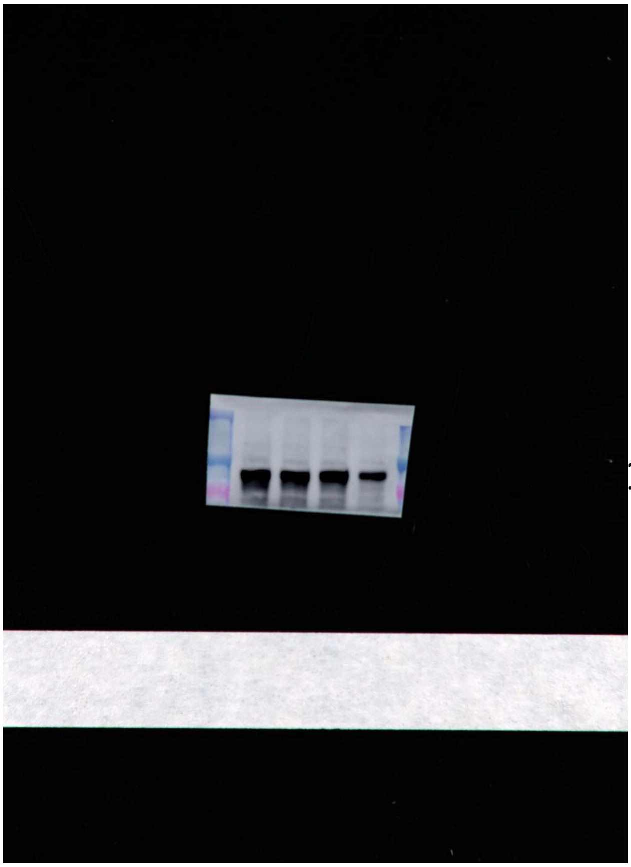

F S10

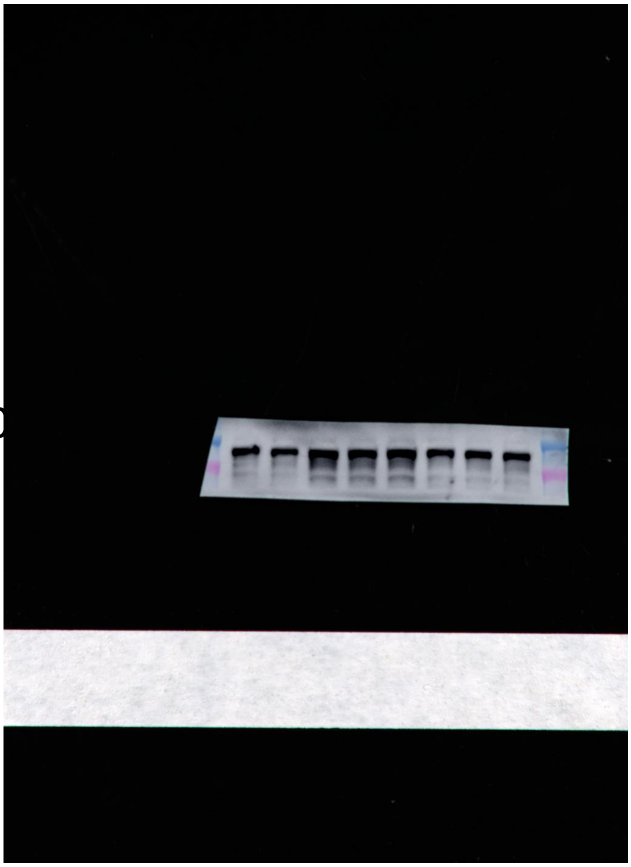

150
100
75

VCAM1

F6

F S10

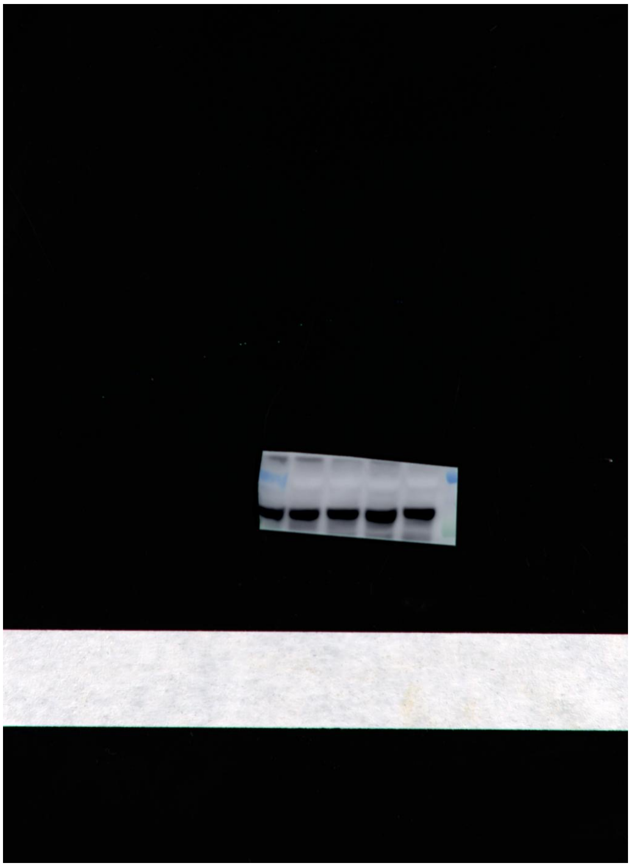

50

37

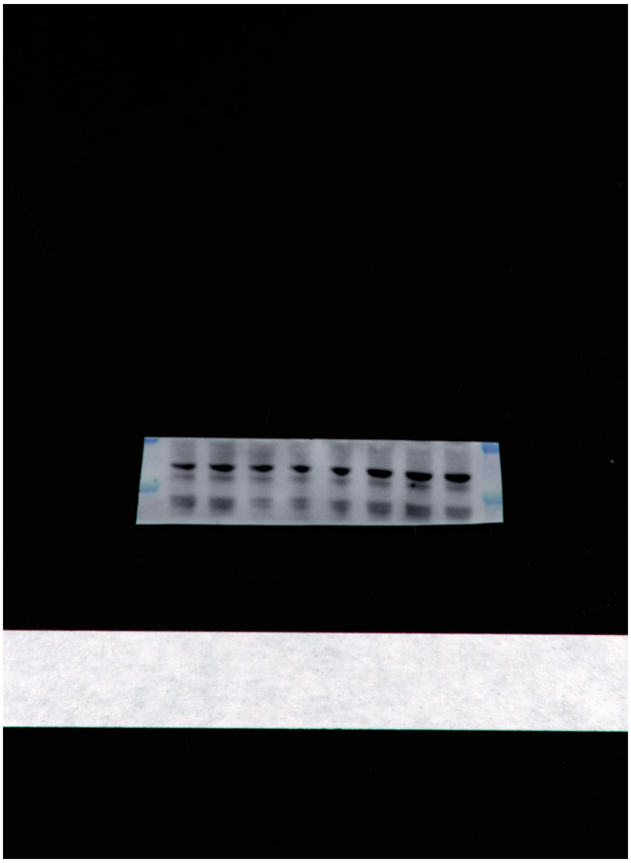

beta-ACTIN
