# Supplementary figures and images for: Refining the optimal CAF cluster marker for predicting TME-dependent survival expectancy and treatment benefits in NSCLC patients
Source: Sci Rep. 2024 Jul 21;14:16766. doi: 10.1038/s41598-024-55375-0 (PMC11271481; doi:10.1038/s41598-024-55375-0)

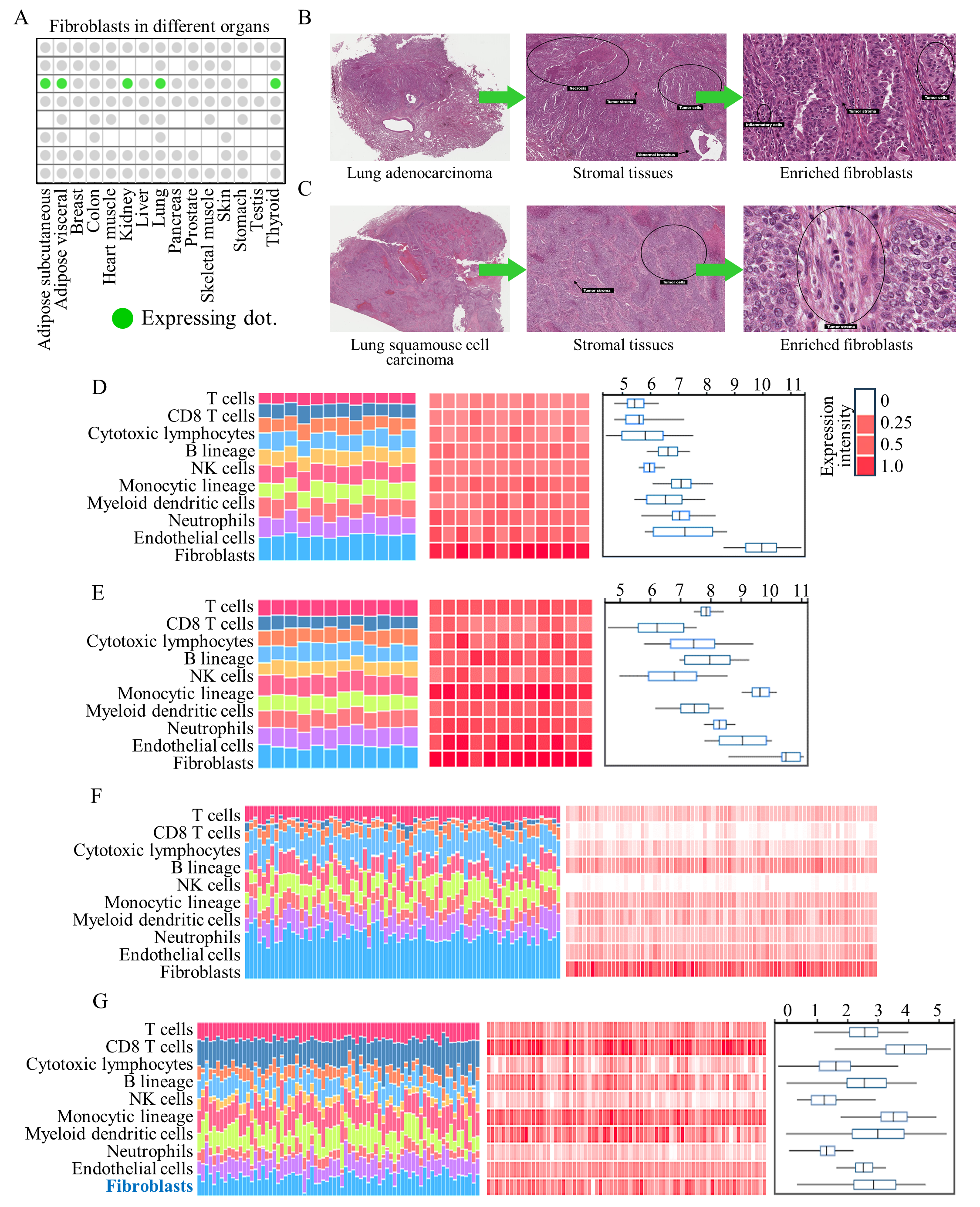

Supplement: Supplementary file 2 — Supplementary Figure S1. [file 41598_2024_55375_MOESM2_ESM.tif]

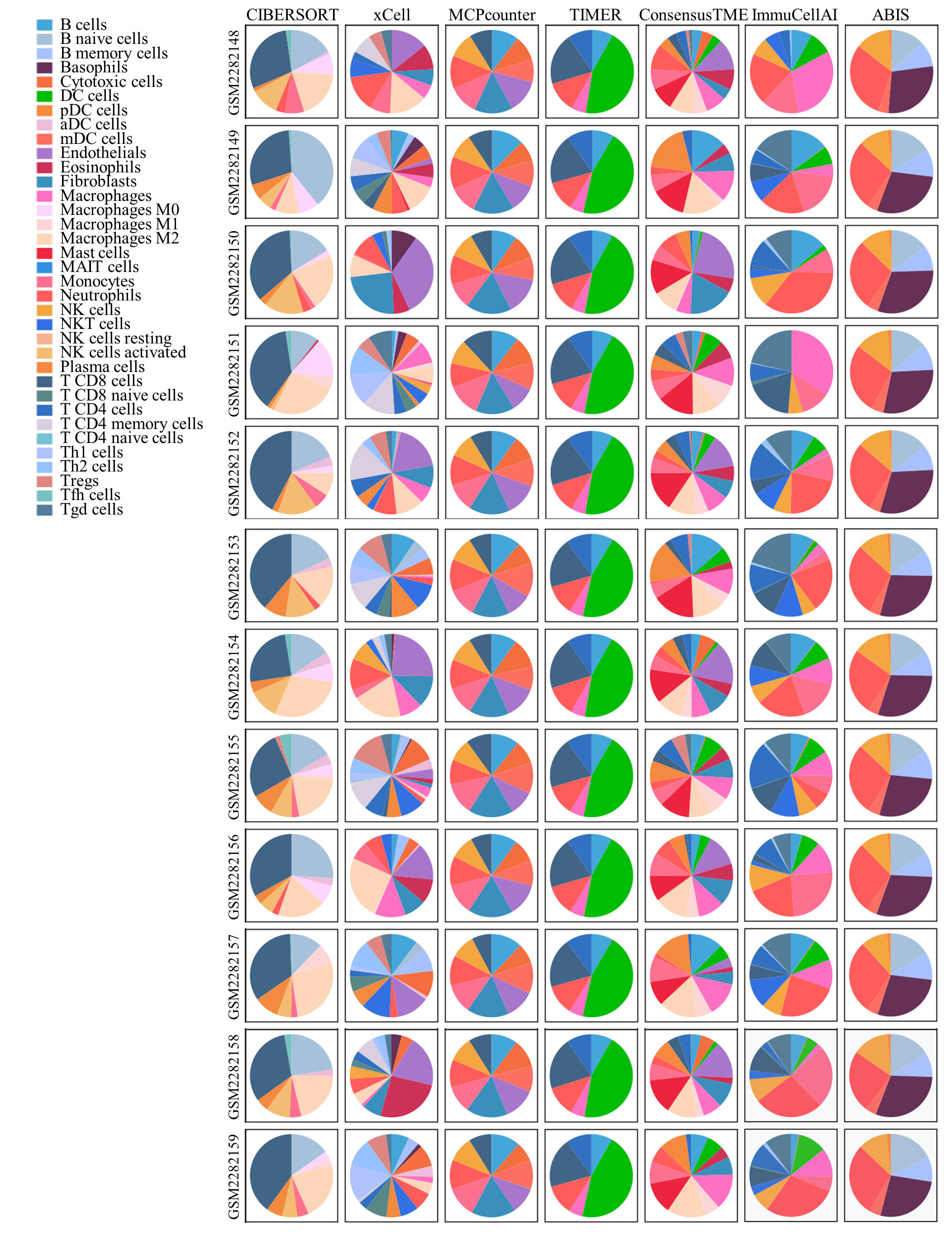

Supplement: Supplementary file 3 — Supplementary Figure S2. [file 41598_2024_55375_MOESM3_ESM.tif]

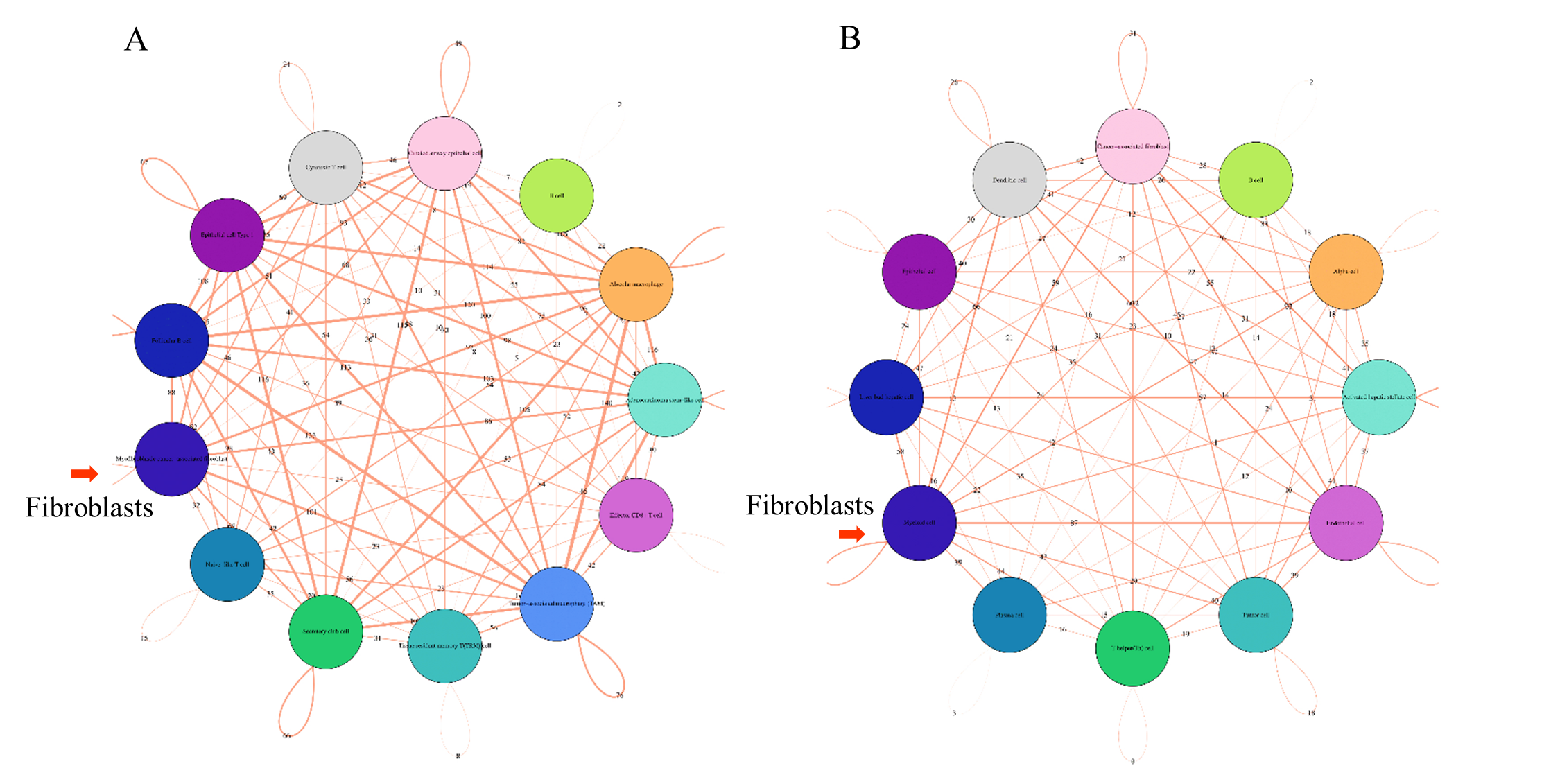

Supplement: Supplementary file 4 — Supplementary Figure S3. [file 41598_2024_55375_MOESM4_ESM.tif]

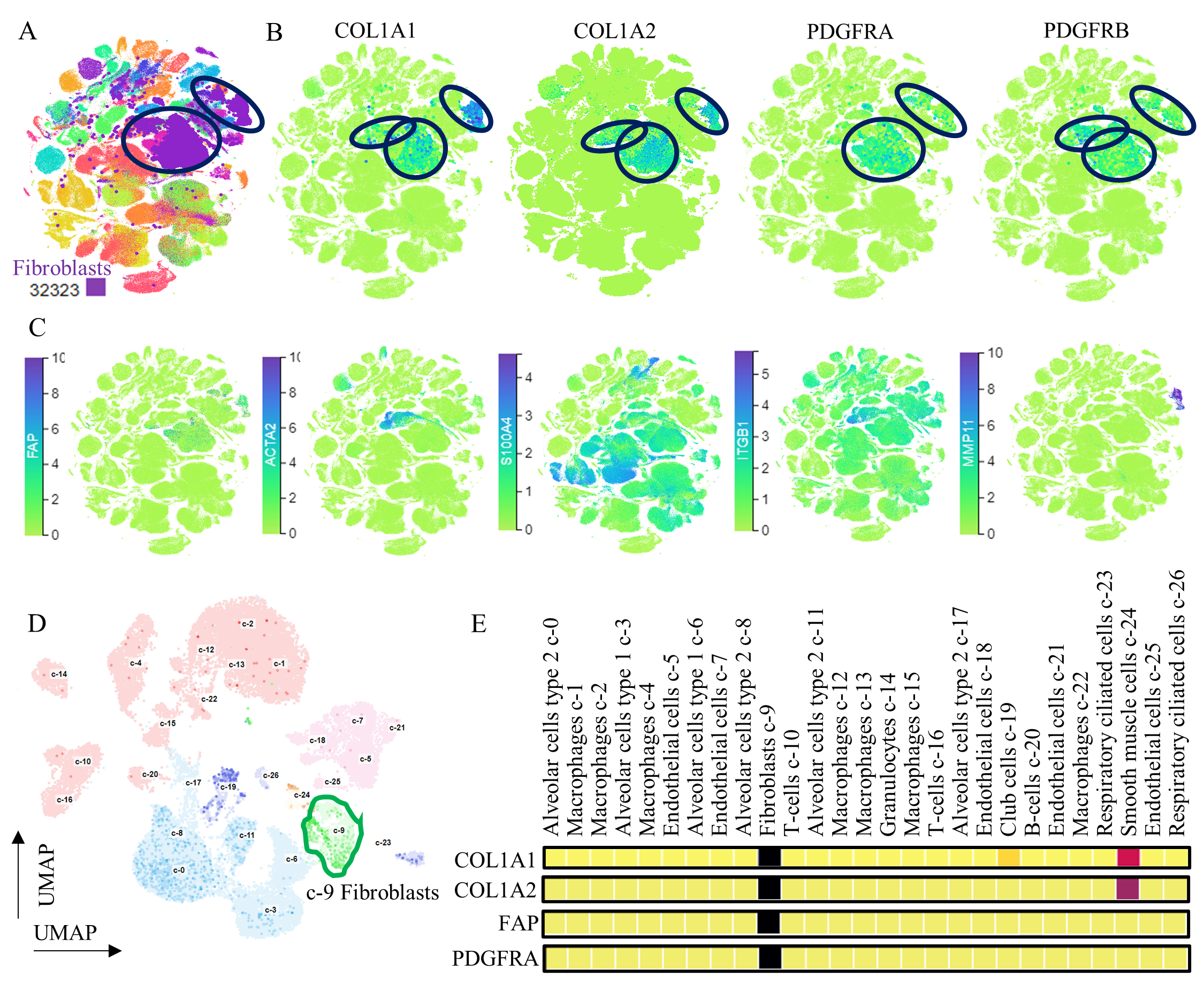

Supplement: Supplementary file 5 — Supplementary Figure S4. [file 41598_2024_55375_MOESM5_ESM.tif]

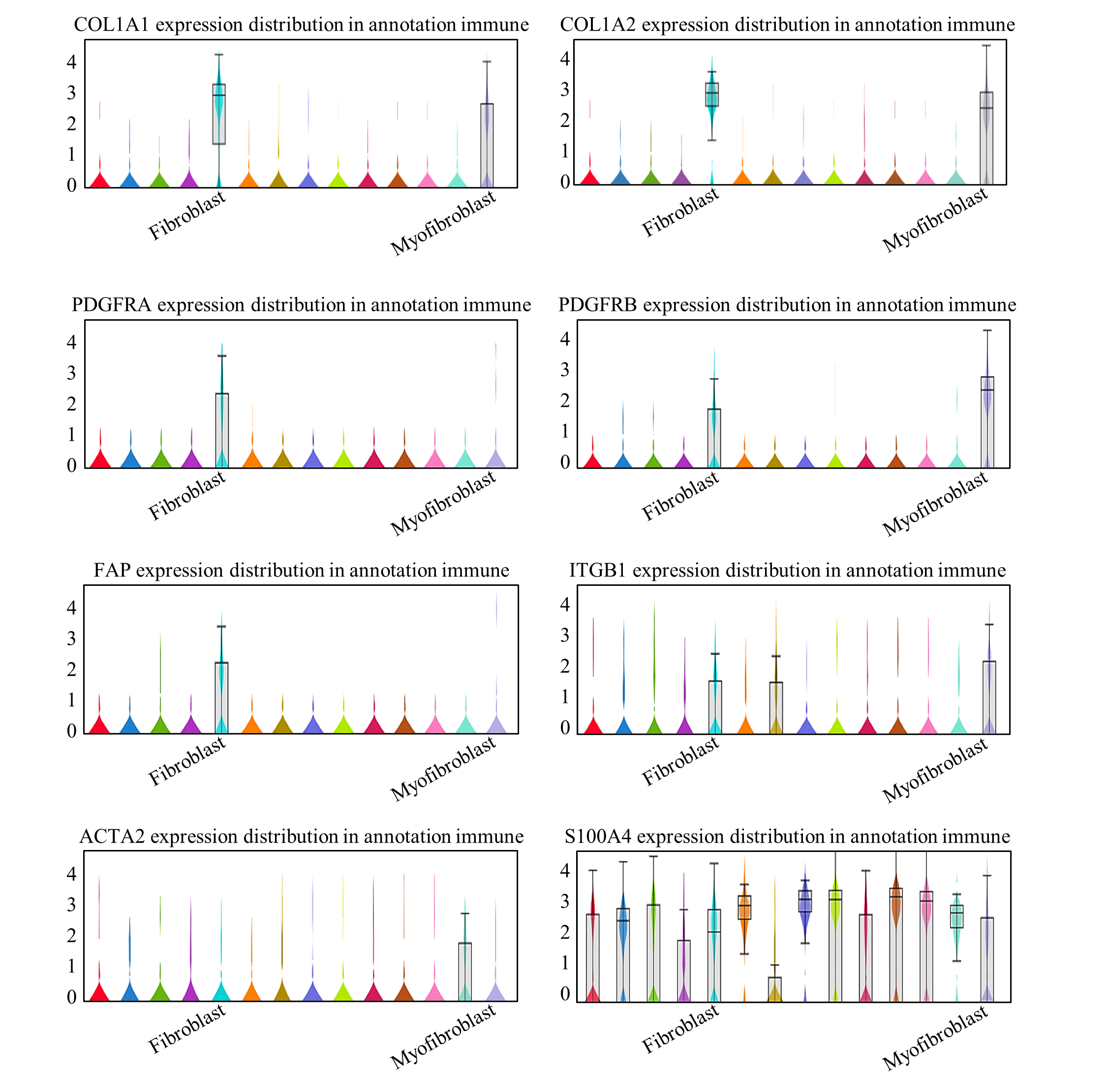

Supplement: Supplementary file 6 — Supplementary Figure S5. [file 41598_2024_55375_MOESM6_ESM.tif]

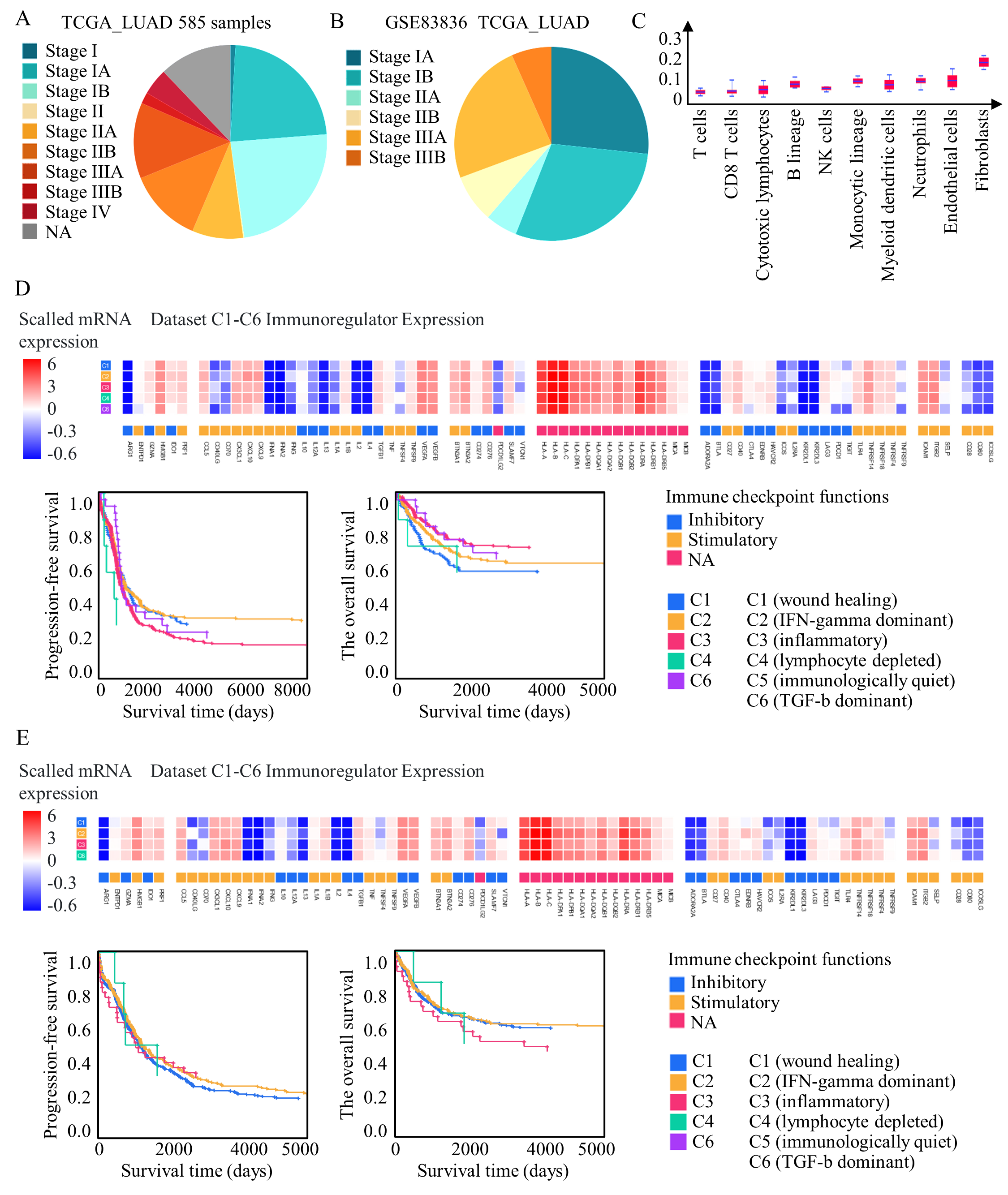

Supplement: Supplementary file 7 — Supplementary Figure S6. [file 41598_2024_55375_MOESM7_ESM.tif]

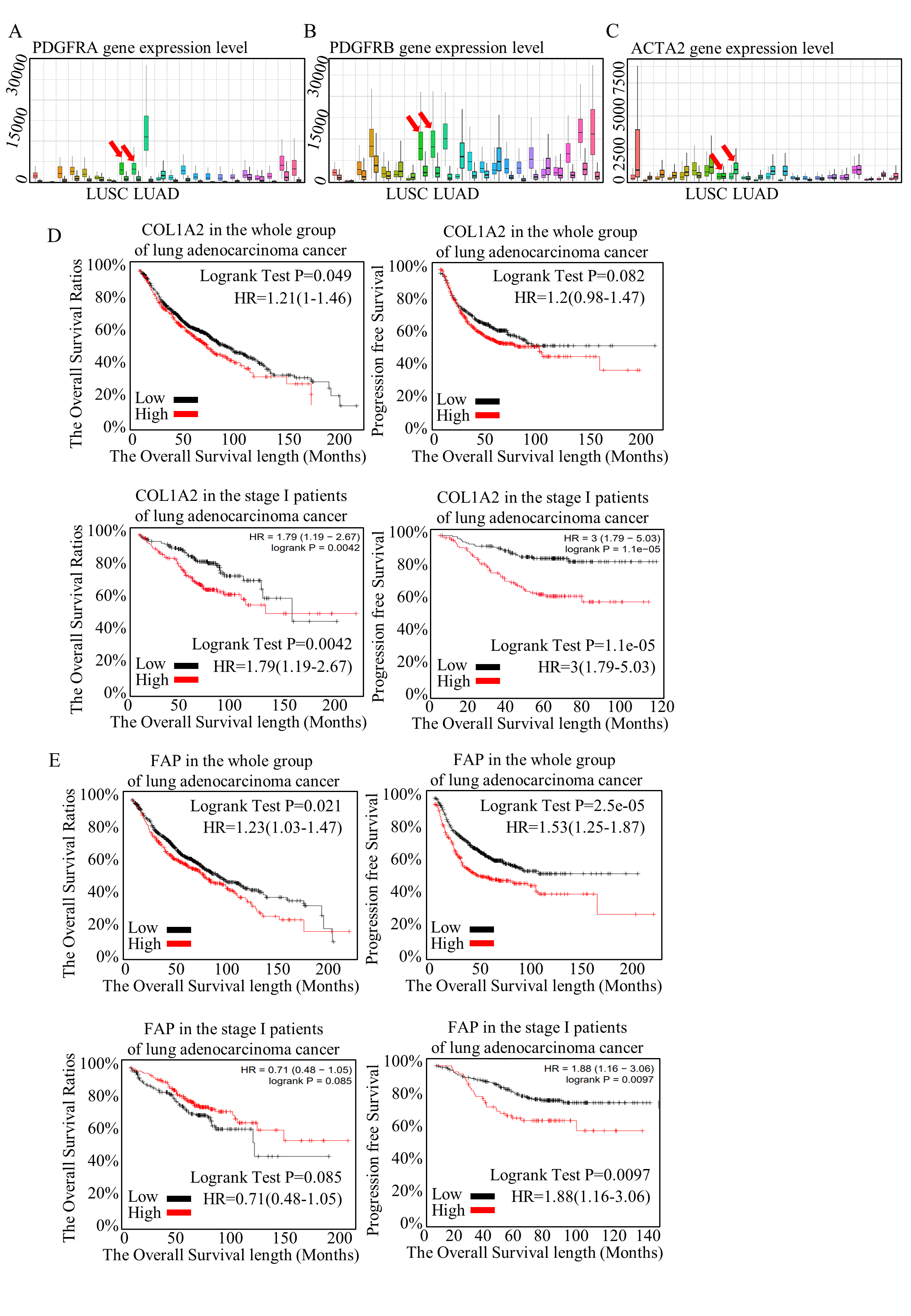

Supplement: Supplementary file 8 — Supplementary Figure S7. [file 41598_2024_55375_MOESM8_ESM.tif]

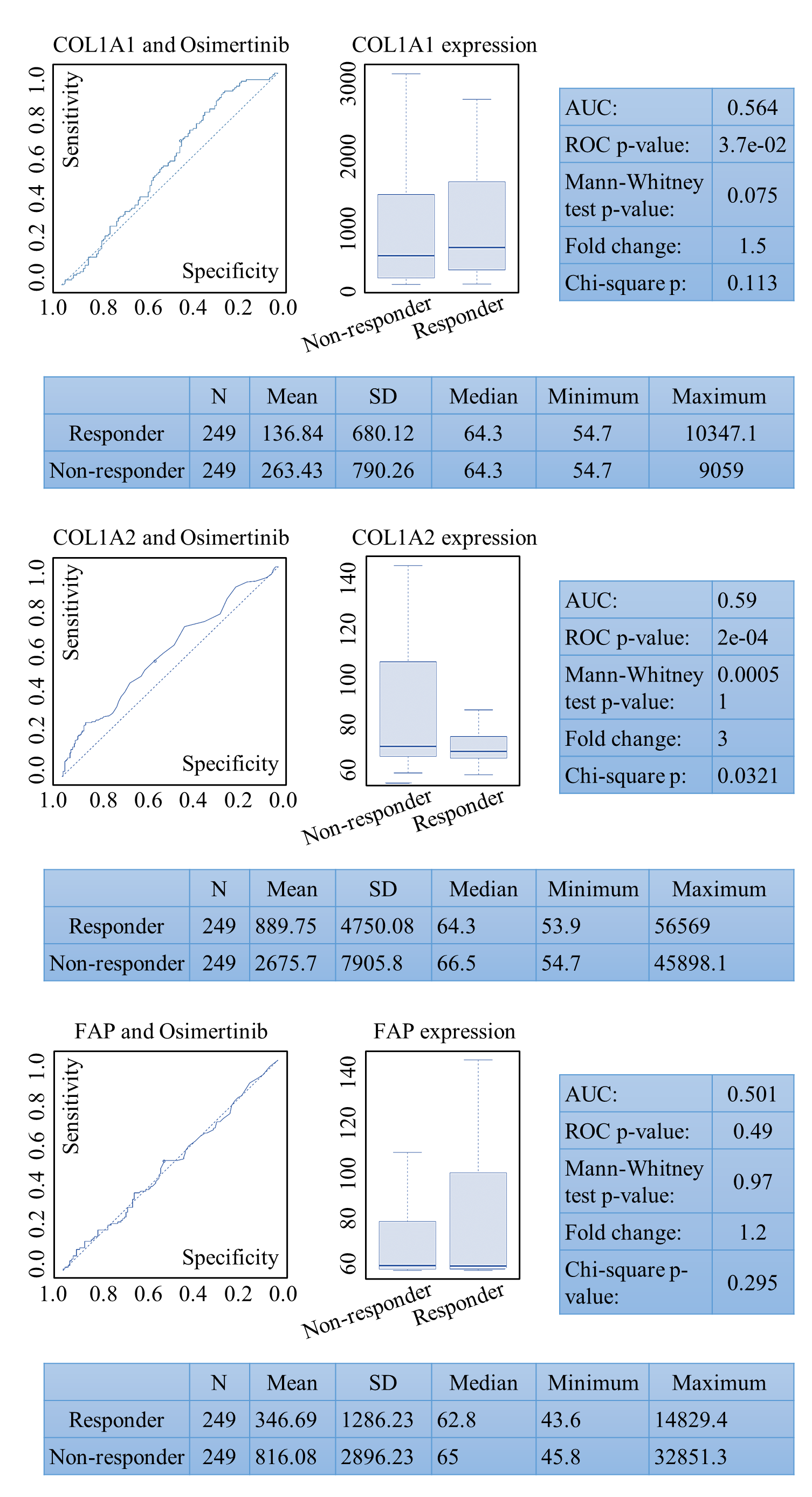

Supplement: Supplementary file 9 — Supplementary Figure S8. [file 41598_2024_55375_MOESM9_ESM.tif]

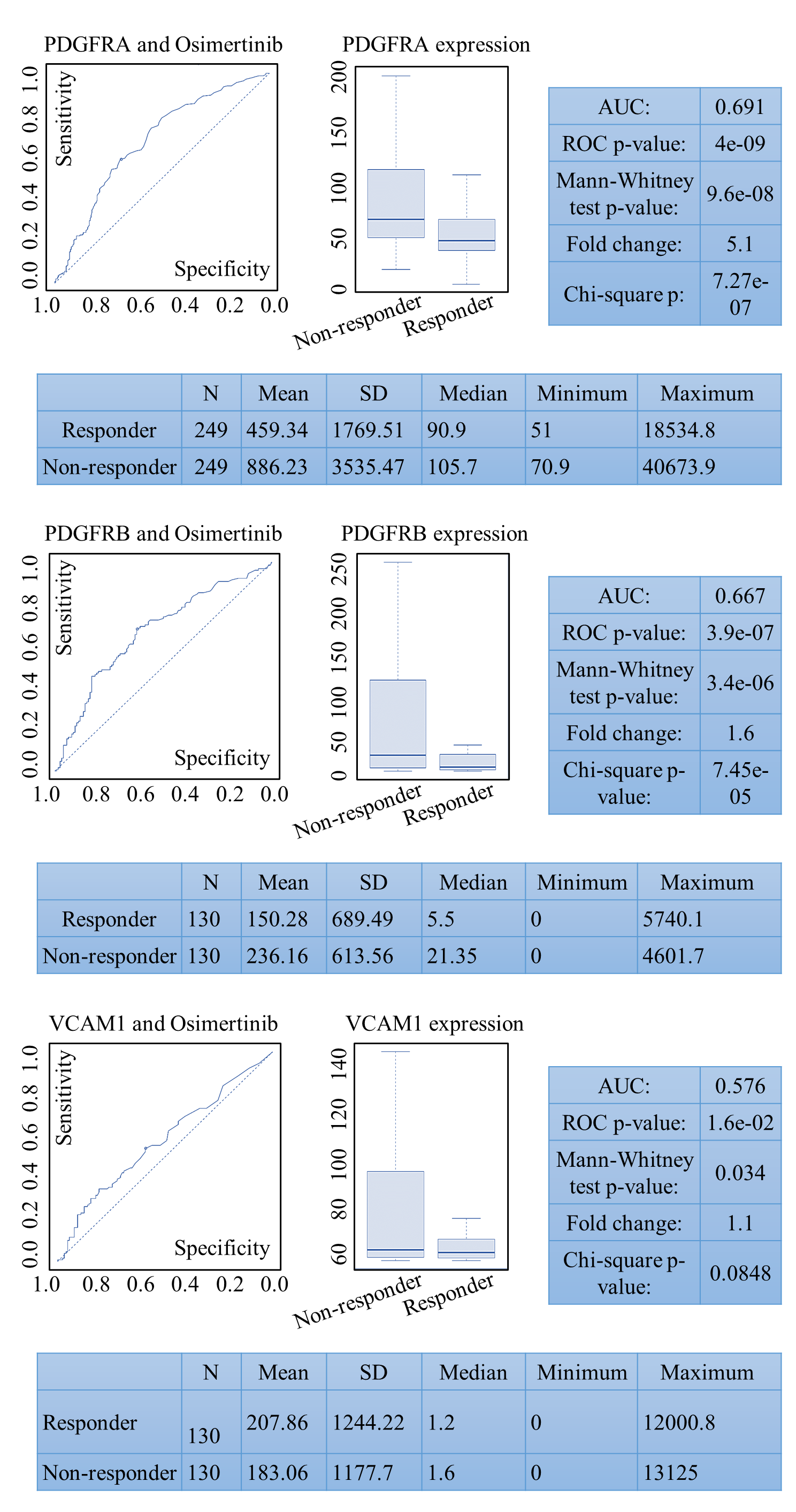

Supplement: Supplementary file 10 — Supplementary Figure S9. [file 41598_2024_55375_MOESM10_ESM.tif]

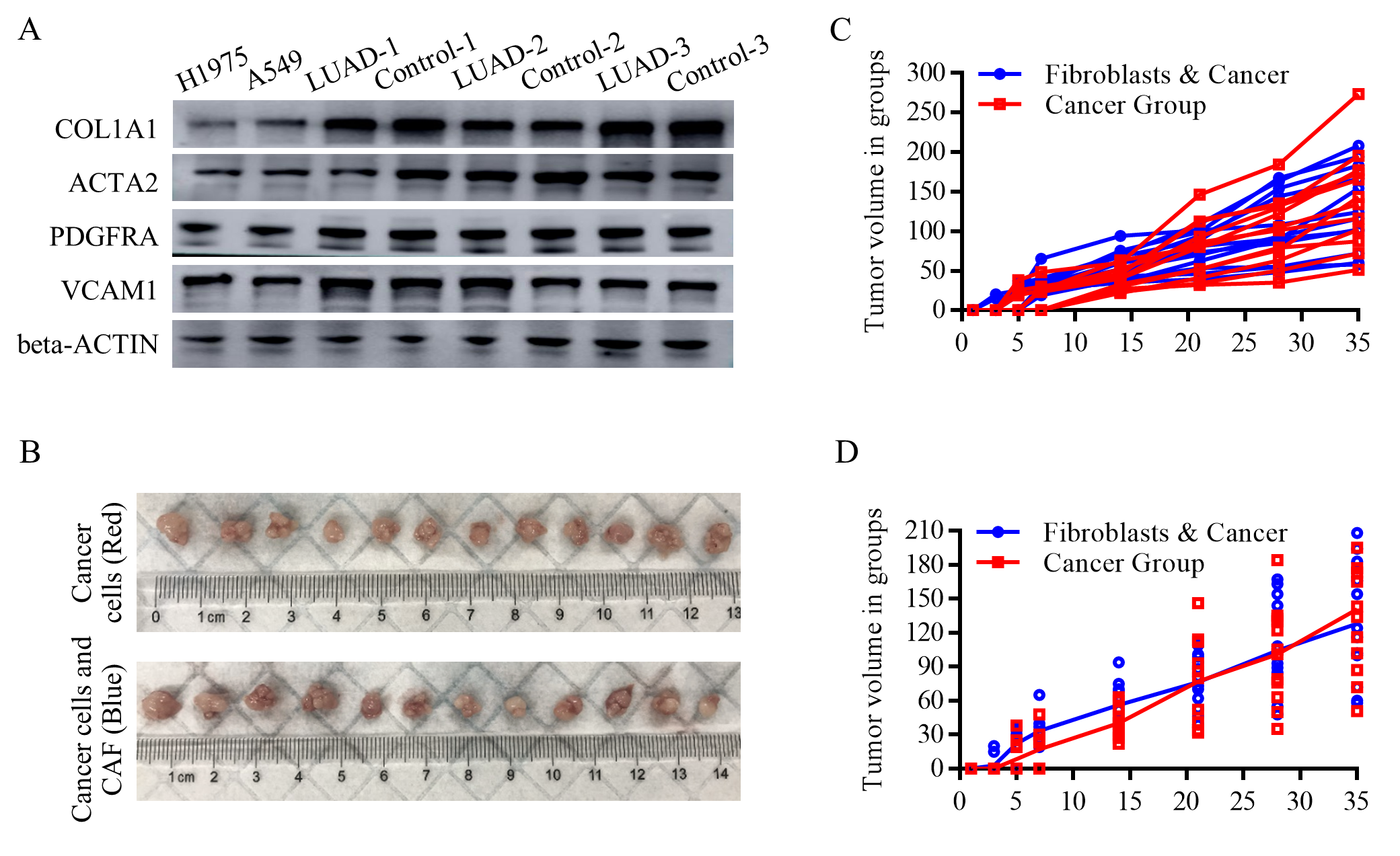

Supplement: Supplementary file 11 — Supplementary Figure S10. [file 41598_2024_55375_MOESM11_ESM.tif]
