# Supplementary material for: Refining the optimal CAF cluster marker for predicting TME-dependent survival expectancy and treatment benefits in NSCLC patients
Source: Sci Rep. 2024 Jul 21;14:16766. doi: 10.1038/s41598-024-55375-0 (PMC11271481; doi:10.1038/s41598-024-55375-0)
Supplement: Supplementary file 12 — Supplementary Legends. [file 41598_2024_55375_MOESM12_ESM.docx]

**Supplemental Figure 1 The landscape of cancer associated fibroblasts**

A. The core cell type table from Protein-Atlas displayed data for a panel of eight cell types that are found in many tissues, and green dots indicate that fibroblast is the core cell type specificity in the indicated tissue. While the grey dots indicated that cell type is present within that tissue, but the selected gene is not predicted to be enriched there. B-C. HE staining of the representative stromal cells’ images were showed. The single cells analysis of GSE85716 (D), GSE118370 (E), and in two TCGA lung cancer data archives (F-G) all revealed the evidently enriched fibroblasts among all TME cells group. The red dots indicated different expression intensity, and different kinds of cells were indicated with different colors, as were indicated in the left line of each panel.

**Supplemental Figure 2 The repertoire of tumors’ TME signatures**

TME consists of many kinds of immune cells and non-immune cells, among these components, CAF was one of the most important partial.

**Supplemental Figure 3 Connections between fibroblasts and other TME subgroups**

Network showed the relationships between fibroblasts (including myofibroblast), and other kinds of TME cells. The numbers of lines and the thickness of lines indicated the close connections.

**Supplemental Figure 4 The sub group of fibroblasts and relevant fibroblasts markers.**

A. The diverse distributing patterns of different TME subgroups were shown. B. The markers of COL1A1, COL1A2, PDGFRA, PDGFRB, represented the distribution of fibroblasts best. C. Many other fibroblast markers are less specific to identify the unique subgroup, and the universal identifying markers of ITGB1, FAP. SOX4, NOTCH3, KLF4, ACTA2, showed either limited or non-specific distribution in whole organs analysis. D-E. Markers of COL1A1, PDGFRA, PDGFRB, and FAP are highly specific in c-9 group of fibroblasts. In detail, COL1A1 is uniquely expressed in fibroblasts with high expression level of 336.7 nTPM, COL1A2 is uniquely expressed in fibroblasts with high expression level of 630.6 nTPM, FAP is uniquely expressed in fibroblasts with high expression level of 630.6 nTPM, PDGFRA is uniquely expressed in fibroblasts with high expression level of 214.0 nTPM.

**Supplemental Figure 5 The sensitivity and specificity of clusters of candidate CAF markers**

COL1A1, COL1A2, PDGFRA, PDGFRB, FAP, ITGB1, ACTA2, S100A4 were all checked in lung cancer stromal cells, and they presented different distribution patterns.

**Supplemental Figure 6 The ratios and functions of fibroblasts helped to predict the survival**

A-B. Two TCGA datasets are enrolled for scRNA and TME signatures analysis. C. Fibroblasts constituted the major part in each LUAD sample dataset. D-E. The fibroblasts were divided on immune-regulator expressing patterns respectively on two TCGA datasets, different fibroblasts clusters indicated different progression survival and overall survival. No significant differences were showed in lung squamous carcinoma, although the CAF clusters constituted the main TME component.

**Supplemental Figure 7 Partial CAF markers cannot differentiate tumor group and normal tissues**

A-C. PDGFRA, PDGFRB, and ACTA2 either failed to be differentially expressed in cancer tissues, or were expressed in relative lower levels. D. COL1A2 did not distinguish the survival differences of overall, progression, and post-progression in the whole groups of lung cancer patients. E. FAP did not define any difference in progression-free of other groups of lung adenocarcinoma.

**Supplemental Figure 8-9 CAF indicated different TKI treatment response**

The roles of CAF markers in predicating TKI therapy of Osimertinib response, and all raw data could be achieved at <http://cis.hku.hk/TISIDB/data_temp/COL1A1_exp_LUSC_isubtype.txt>, and <http://cis.hku.hk/TISIDB/data_temp/COL1A1_exp_LUAD_isubtype.txt>, and <http://cis.hku.hk/TISIDB/browse.php?gene=COL1A2>. COL1A1 and COL1A2 showed the best diagnosing accuracy, and the survival predicating sensitivity, and the therapy response specificity.

**Supplemental Figure 10 CAF markers and functions evaluation**

A. The CAF markers in lung cancer cell lines, in lung cancer tissues, and in adjacent lung tissues. B-D. Tumor growth in Vivo study, and the plots image, together with scatter figure were provided to go with Figure 6E and 6F.
